# Colonization of gut microbiota by plasmid-carrying bacteria is facilitated by evolutionary adaptation to antibiotic treatment

**DOI:** 10.1038/s41396-021-01171-x

**Published:** 2021-12-13

**Authors:** Peng Zhang, Daqing Mao, Huihui Gao, Liyang Zheng, Zeyou Chen, Yuting Gao, Yitao Duan, Jianhua Guo, Yi Luo, Hongqiang Ren

**Affiliations:** 1 College of Environmental Sciences and Engineering, Nankai University, Tianjin, 300350 China; 2 State Key Laboratory of Pollution Control and Resource Reuse, School of the Environment, Nanjing University, Nanjing, 210046 China; 3 School of Medicine, Nankai University, Tianjin, 300071 China; 4 Australian Centre for Water and Environmental Biotechnology, The University of Queensland, St. Lucia, Brisbane, QLD 4072 Australia

**Keywords:** Antibiotics, Molecular evolution

## Abstract

Multidrug-resistant plasmid-carrying bacteria are of particular clinical concern as they could transfer antibiotic resistance genes to other bacterial species. However, little is known whether evolutionary adaptation of plasmid-carrying bacteria after long-term antibiotic exposure could affect their subsequent colonization of the human gut. Herein, we combined a long-term evolutionary model based on *Escherichia coli* K-12 MG1655 and the multidrug-resistant plasmid RP4 with in vivo colonization experiments in mice. We found that the evolutionary adaptation of plasmid-carrying bacteria to antibiotic exposure facilitated colonization of the murine gut and subsequent plasmid transfer to gut bacteria. The evolved plasmid-carrying bacteria exhibited phenotypic alterations, including multidrug resistance, enhanced bacterial growth and biofilm formation capability and decreased plasmid fitness cost, which might be jointly caused by chromosomal mutations (SNPs in *rpoC*, *proQ*, and *hcaT*) and transcriptional modifications. The upregulated transcriptional genes, e.g., type 1 fimbrial-protein pilus (*fimA* and *fimH*) and the surface adhesin gene (*flu*) were likely responsible for the enhanced biofilm-forming capacity. The gene *tnaA* that encodes a tryptophanase-catalyzing indole formation was transcriptionally upregulated, and increased indole products participated in facilitating the maximum population density of the evolved strains. Furthermore, several chromosomal genes encoding efflux pumps (acriflavine resistance proteins A and B (*acrA, acrB*), outer-membrane protein (*tolC*), multidrug-resistance protein (*mdtM*), and macrolide export proteins A and B (*macA*, *macB*)) were transcriptionally upregulated, while most plasmid-harboring genes (conjugal transfer protein (*traF*) and (*trbB*), replication protein gene (*trfA*), beta-lactamase TEM precursor (*bla*_*TEM*_), aminoglycoside 3'-phosphotransferase (*aphA*) and tetracycline resistance protein A (*tetA*)) were downregulated. Collectively, these findings demonstrated that evolutionary adaptation of plasmid-carrying bacteria in an antibiotic-influenced environment facilitated colonization of the murine gut by the bacteria and plasmids.

## Introduction

Antimicrobial resistance (AMR) has been recognized as a critical global-health crisis that will cause a predicted 10 million deaths by 2050 if no action is taken now [1]. Antibiotic-resistant bacteria can spread across humans, animals, and the environment [2]. In particular, plasmid-carrying bacteria represents a very common form of multidrug-resistant (MDR) pathogen [3, 4]; and they are extremely prevalent in both clinical and environmental settings. Colonization is a crucial step of MDR pathogen transmission from the environment toward humans. Only a subset of exogenous bacteria manages to colonize animal or human gut microbiota depending on their colonization ability [5].

Recently, an increasing number of studies have focused on the evolutionary dynamics and fates of plasmid-carrying bacteria (plasmid cost *versus* compensation). Some in vitro studies have documented that most plasmids reduce bacterial fitness in the absence of selection for plasmid-encoded traits [6–8]. However, the cost of plasmid carriage can be alleviated by compensatory evolution resulting in genetic modifications [9–11]. For example, mutations to plasmid-harboring gene *trfA* that encodes plasmid replication initiation protein alleviate plasmid burden by lowering plasmid replication capacity [12], and mutations to chromosomal gene, including *Xpd*/*Rad3*, *UvrD*, and *RpoB* also participated in ameliorating plasmid cost via interacting with DNA helicases [13]. In addition, several bacterial phenotypes (e.g., antibiotic resistance, growth, and biofilm-forming capacity) might be associated with bacterial colonization ability because they confer extended survival capability. The properties of improved growth and biofilm-forming capacity can help exogenous bacteria successfully compete with other gut bacteria to colonize the gastrointestinal tract [5, 14].

Although previous studies have explored the evolutionary dynamics of plasmid-carrying bacteria under antibiotic pressure, most findings have come from in vitro experiments. In particular, whether evolutionary adaptation of plasmid-carrying bacteria to antibiotic exposure could affect their subsequent colonization of the gut is not known. To fill this knowledge gap, we combined a long-term evolutionary model based on *Escherichia coli* K-12 MG1655 and the multidrug resistance plasmid RP4 with in *vivo* colonization experiments in mice. The plasmid-carrying bacteria were cultured for 150 days (~1000 generations) in the presence of antibiotics. We systematically compared bacterial growth capacities, biofilm formation, antibiotic resistance, and plasmid fitness cost in plasmid-carrying bacteria cultured with or without antibiotic exposure. In addition, genetic alterations to these plasmid-carrying bacterial isolates were analyzed using whole-genome deoxyribonucleic acid (DNA) sequencing. The phenotypic modifications and subsequent colonizing the murine gut of the evolved strains were explored. The growth rate of the evolved strains was also investigated in both in vitro experiment and in *vivo* murine livers and spleens. This study provides insights into mammalian-gut colonization of exogenous plasmid-carrying bacteria that were exposed to antibiotics, highlighting the transmission risk of antibiotic resistance encoded by plasmids from the environment toward humans.

## Materials and Methods

### Strains, culture conditions, and evolutionary experiments with plasmid-carrying bacteria

The plasmids and bacterial strains used in this study are listed in Table S1. Plasmid-free *E. coli* K12 MG1655 strain was treated as parental hosts (H), while the conjugative plasmid RP4 (including beta-lactamase TEM precursor [*bla*_*TEM*_], tetracycline resistance protein A [*tetA*] and aminoglycoside 3′-phosphotransferase A [*aphA*]) [15] was introduced into the H strains via electroporation to obtain ancestral strains (A:H:p) as previously described [16]. In the evolutionary experiments, all bacterial strains were grown in Luria–Bertani (LB) broth in 96-well microplates at 37 °C and were shaken at 200 rpm.

**Fig. 1:**
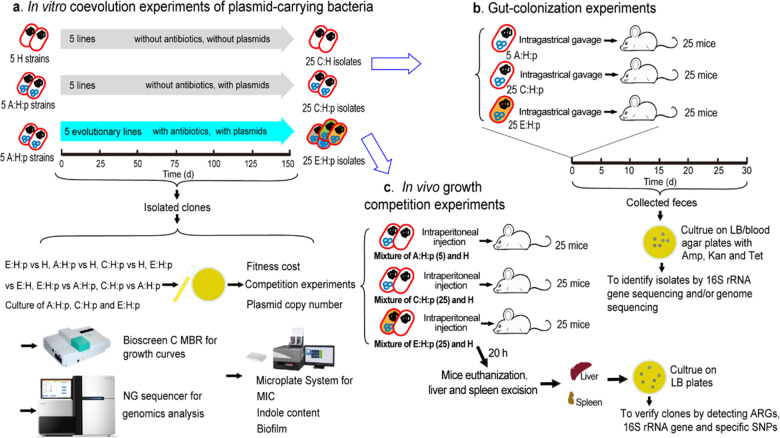
Evolutionary-experiment design and methodologies. **a** In vitro coevolution experiments of plasmid-carrying bacteria. Plasmid-free *E. coli* K12 MG1655 strains were treated as parental hosts (H), while original RP4-carrying *E. coli* K12 MG1655 strains were treated as ancestral bacterial hosts with plasmids (A:H:p). These evolved strains (E:H:p) were obtained by evolving A:H:p strains under antibiotic exposure (ampicillin: 10 mg/L; kanamycin: 5 mg/L) for 150 days. Control strains (C:H or C:H:p) were obtained by culturing H or A:H:p strains without antibiotic exposure for 150 days. Each A:H:p strain was considered to be one evolutionary line. **b** Gut colonization experiments of A:H:p, C:H:p, and E:H:p strains. **c** In vivo competition experiments of A:H:p, C:H:p, and E:H:p strains against H strains in mice through intraperitoneal injection.

As shown in Fig. 1, in our long-term evolutionary experiments we treated five independent A:H:p strains representing five evolutionary lines with two antibiotics (10 mg L^−1^ ampicillin and 5 mg L^−1^ kanamycin) to obtain evolved plasmid-carrying bacteria (E:H:p). Lines of both H and A:H:p strains were grown in parallel in the absence of antibiotic treatment to obtain bacterial host control (C:H) and plasmid-carrying bacterial control (C:H:p); these lines were grown five times to enable five replications of the experiments. The concentration of antibiotics used in these experiments was just slightly higher than the MIC of plasmid-free H strains and lower than that of A:H:p strains. We transferred these evolutionary cultures (1:100) into fresh media and antibiotics every 24 h for 150 days, producing about 1000 bacterial generations. During the evolutionary period, 10–20 evolved strains (evolved bacterial hosts with plasmids) were randomly isolated at different time points (days 25, 50, 100, and 150) and used to monitor phenotypic changes. In addition, we randomly picked five end-point isolates from each line for phenotypic and genotypic analyses of growth curve measurement, biofilm formation, antibiotic resistance, and DNA sequencing. Every 10 days throughout the experiment, we collected 100 μL samples of whole evolutionary lines and stored them in 25% glycerol at −80° C. Then, we performed several assays and experiments on E:H:p strains to estimate in vitro fitness cost, growth competition, and growth curve (detailed in Text S1 in the Supplementary information); MIC measurement, plasmid copy number determination (PCN; detailed in Text S2), indole content (detailed in Text S3), bacterial biofilm formation (detailed in Text S4) and plasmid conjugative transfer rate (detailed in Text S5); and gut colonization and in vivo growth competition in order to determine the evolutionary phenotypes. Bacterial whole-genome sequencing (WGS), quantitative reverse transcription-polymerase chain reaction (RT-qPCR) and Sanger sequencing were employed to decipher the evolutionary genotypic modifications to E:H:p strains (details shown below). Also, we cured the plasmid from E:H:p strains (detailed in Text S6) and performed the allelic reconstructions for SNPs (e.g., *proQ*, *hcaT*, and *rpoC*) in WT *E. coli* strains (detailed in Text S7) to dissect the influence of these mutations in the altered phenotypes of E:H:p strains. To validate whether the presence of the plasmid is necessary for the evolutionary adaptation of the plasmid-carrying strains, we evolved the plasmid-free H strains to obtain the control evolved H (C:E:H) strains under antibiotic exposure (2 mg L^−1^ ampicillin and 1 mg L^−1^ kanamycin) for 30 days. After that, WGS (three strains were randomly selected) was applied for plasmid-free H (C:E:H) strains to explore nonsynonymous mutations.

### The competition assays

To estimate the bacterial fitness in vitro, we evaluated the fitness (RF) of E:H:p, A:H:p and C:H:p strains by comparing E:H:p with H, A:H:p with H, and C:H:p with H. Briefly, pure cultures of these strains grown in LB at 37 °C for 12 h were separately diluted to 5 × 10^8^ CFU mL^−1^; then, we mixed E:H:p with H, A:H:p with H, and C:H:p with H at ratios of 1:1. Afterward, all three mixtures were diluted 1000-fold into fresh non-selective LB media, incubated at 37 °C and spun at 200 rpm. Next, we diluted the cultures appropriately and spread them on LB plates with or without antibiotics (ampicillin, kanamycin, and tetracycline mixture). Bacteria were counted at 0 and 24 h, and the RF of the plasmid-carrying strains was calculated as a ratio of Malthusian parameters [17]:

$$RF = \frac{{\ln (\frac{{N_{{{{{{{{\mathrm{final}}}}}}}},\;{{{{{{{\mathrm{p}}}}}}}} + }}}{{N_{{{{{{{{\mathrm{initial}}}}}}}},{{{{{{{\mathrm{p}}}}}}}} + }}})}}{{\ln (\frac{{N_{{{{{{{{\mathrm{final}}}}}}}},\;{{{{{{{\mathrm{p}}}}}}}} - }}}{{N_{{{{{{{{\mathrm{initial}}}}}}}},{{{{{{{\mathrm{p}}}}}}}} - }}})}}$$

where *N*_initial_ and *N*_final_ represented the initial and final numbers of strains, *P+* stood for plasmid-carrying strains (E:H:p, A:H:p, and C:H:p) and *P−* stood for the ancestral plasmid-free strain (H). RF score >1 indicated that the E:H:p strains had a selective advantage over the H strain or vice versa.

To examine the growth advantages of E:H:p strains against A:H:p or C:H:p strains, we used the ceftriaxone-resistant phenotype acquired by E:H:p strains in evolutionary adaptation. In brief, all three strains were grown in LB at 37° C for 12 h and then separately diluted to 5 × 10^8^ CFU mL^−1^. We mixed the diluted A:H:p or C:H:p strains (5 × 10^8^ CFU mL^−1^) with E:H:p (5×10^8^ CFU mL^−1^) at a 1:1 ratio, further diluted the mixture 1000-fold in fresh non-selective LB media, incubated it at 37 °C and spun it at 200 rpm for 24 h. Afterward, the cultures were appropriately diluted and spread on LB plates with or without the addition of ceftriaxone, and bacterial CFU were enumerated at 0 and 24 h. The RF of E:H:p to A:H:p or C:H:p was calculated using the above-described formula for RF. We further verified the E:H:p clones selected from the plates via PCR and Sanger sequencing to detect specific single-nucleotide polymorphism (SNP) markers on chromosomal RNA polymerase subunit β′ (*rpoC*) acquired by evolutionary mutation of E:H:p.

### Minimal inhibitory concentration measurement

To test changes of antibiotic resistance after long-term evolution, MIC was measured as previously described [18]. We grew five isolates per line overnight in 2 mL LB broth. Then, the saturated cultures (20 µL) of these isolates were seeded into 2 mL fresh LB and subcultured to an optical density (OD) of ~0.5 at 600 nm. We subsequently added the cultures into 96-well plates containing a log2 serial dilution of antibiotics (ampicillin, kanamycin, tetracycline, erythromycin, streptomycin, and ceftriaxone), with an initial density of 5 × 10^5^ CFU mL^−1^. After 24 h of incubation at 37 °C, the cultures were shaken and recorded at OD_600_.

### Plasmid copy number determination

We determined the RP4 PCN in A:H:p, C:H:p and E:H:p strains via droplet digital PCR (ddPCR) in accordance with a previous study [19]. Details of quantification and amplification conditions are provided in the Supplementary Information (Text S2).

### Genome sequencing and bioinformatic analysis

To estimate the occurrence of genomic mutations during evolutionary adaptation to antibiotic stress, we extracted bacterial genomic DNA (gDNA) from the five E:H:p strains isolated at the end of our evolutionary experiments and from control lines (5 A:H:p, 5 C:H:p, and 3 H strains) using a QIAGEN DNA Mini Kit (QIAGEN, Hilden, Germany) per manufacturer’s instructions. The quality and integrity of gDNA were assessed using agarose gel electrophoresis; specifically, 1 μl of DNA extract was analyzed in a 1% agarose gel with non-toxic dye (US Everbright; Suzhou, China), and concentration was determined using a Nanophotometer N60 (Implen, Munich, Germany). DNA sequencing was performed on an MiSeq platform (Illumina, Inc., San Diego, CA, USA) at Beijing Novogene Technology Co., Ltd. (Beijing, China). We trimmed sequencing adapters and low-quality bases using FastQC software version 0.11.9 [20]. The clean reads were further mapped to the genomic sequences of *E. coli* K-12 MG1655 (NC_000913.3) and RP4 (L27758.1) using Burrows–Wheeler Aligner (BWA)–MEM software version 0.7.17 [21]. We detected SNPs and indels using SAMtools software version 1.10 [22] and annotated them using Sorting Intolerant From Tolerant (SIFT) 4G software and SnpEff software version 4.3 (genetic variant annotation and functional effect prediction toolbox) [23, 24]. Insertion sequences (ISs) were identified using custom scripts and Integrative Genomics Viewer software version 9.1.0 [25], and large genome-wide structural variants were detected using BreakDancer software version 1.1 [26]. We used PCR and Sanger sequencing to verify bacterial genomic mutations in E:H:p clones.

### Quantitative reverse transcription-polymerase chain reaction

Messenger RNA (mRNA) gene expression was assessed via RT-qPCR using 2 × TB Green Premix (TaKaRa, Japan) per manufacturer’s protocol. We prepared total RNA from E:H:p or A:H:p strains using a TRIzol Reagent Kit (Invitrogen Corp., Carlsbad, CA, USA). RNA concentration was measured on the Nanophotometer N60, and its integrity was validated by agarose gel electrophoresis, followed by complementary DNA (cDNA) synthesis using a PrimeScrip RT Reagent Kit with gDNA Eraser (TaKaRa). For qPCR, we used the chromosomal marker L-idonate and D-gluconate transporter (*idnT*) as the reference gene. The conditions for RT-qPCR are detailed in Text S3 in the Supplemental Methods. The relative expression level was determined using the comparative ^C^_T_ (^ΔΔC^_T_) method [27]. The primers used are listed in Table S2.

### In vivo animal experiments

#### Gut colonization assays

The animal experiments in this study were approved by the Ethics and Clinical Research Committee of Nankai University (Tianjin, China; Project No. IRM-DWLL-2016121). We obtained 80 C57BL/6 female mice (age 6–7 weeks) from Beijing Hfk Bioscience Co., Ltd. (Beijing, China) and housed them in specific-pathogen-free (SPF) facilities. Mice were randomly divided into 16 groups (five per group) and allowed sterilized antibiotic-free water and pelleted food *ad libitum*. After 1 week of acclimatization, each mouse was caged individually, and cage bedding was changed daily. We individually suspended the five lines of A:H:p, C:H:p, and E:H:p strains in phosphate-buffered saline (PBS) solutions and administered 200 µL of each suspension (1 × 10^10^ CFU mL^−1^) intragastrically to mice (as shown in Fig. 1). One control group of mice was treated with PBS solution, five groups of mice were inoculated with E:H:p suspension, five groups were inoculated with A:H:p suspension, and a final five groups of mice were inoculated with the control C:H:p suspension. Fresh fecal samples were collected daily and stored at −20° C before further analysis. Starting on day 20, we administered an antibiotic cocktail (ampicillin, 500 mg L^−1^; kanamycin, 250 mg L^−1^) to all mice through drinking water until the end of the study. To investigate the effects of antibiotic stress on bacterial gut colonization, we chose the high antibiotic concentrations based on their consideration of the absorbability and bioavailability. To accurately determine the minimum concentration of target bacteria, we set the limit of detection for the culture-based methods employed in the present research at 10 CFU g^−1^ feces for A:H:p or C:H:p strains. PCR did not detect any RP4 plasmid in the fecal samples collected before the administration of bacteria.

#### Isolation and verification of RP4-carrying strains from murine feces

We used a blood agar plate with 100 mg L^−1^ ampicillin, 50 mg L^−1^ kanamycin, and 25 mg L^−1^ tetracycline to count and isolate RP4-carrying strains that were intragastrically administered and gut-resident bacteria from murine feces. These RP4-carrying isolates were further verified using multiplex primers (origin of replication [*OriV*], *bla*_*TEM*_ and *tetA*), and we used Sanger sequencing to target chromosome-carried *rpoC* in order to ensure that the E:H:p strains were isolated from the murine gut in this experiment. At the end of the experiment, we randomly chosen ten colonies from the fecal sample of each mouse (total mice number of 25) that was administrated intragastrically with E:H:p strains. In total 250 isolates were collected, and Sanger sequencing was performed on 16S ribosomal RNA (rRNA; 27F and 1492R) to determine bacterial species.

#### In vivo competition assays

To determine the competitive index in vivo, we compared the growth Of E:H:p with H, C:H:p with H, and A:H:p with H in mouse livers and spleens. We diluted the overnight cultures of the E:H:p, C:H:p, A:H:p, and H strains and then mixed E:H:p with H, C:H:p with H, and A:H:p with H at ratios of 1:1 to obtain a 250 µl mixture of 1 × 10^9^ CFU mL^−1^ in PBS. All three mixtures were injected intraperitoneally into mice. After 20 h of incubation, mice were anesthetized with isoflurane and then decapitated. Next, their livers and spleens were aseptically extracted, weighed, and homogenized in ice-cold LB medium in a Mixer Mill dismembrator (Retsch GmhB, Haan, Germany), and then the mixtures were spread on LB plates with or without antibiotics (ampicillin, kanamycin, and tetracycline mixture). Finally, we counted the CFUs of the A:H:p, C:H:p, E:H:p, and H strains in tissue extracts and calculated competitive index in vivo using the CFU ratio of A:H:p, C:H:p, E:H:p strains to H strains.

### Statistical analysis

Data were expressed as mean ± standard deviation (SD). We prepared and calculated averages and SDs using GraphPad Prism version 8.0 (GraphPad Software, Inc., San Diego, CA, USA) and analyzed data using SPSS version 24.0 (IBM Corp., Armonk, NY, USA). Student’s *t* tests and one-way analysis of variance (ANOVA) were performed for group comparisons, and *p* values were corrected using the Benjamini–Hochberg method [28]. *p* < 0.05 was considered statistically significant. All experiments were conducted in triplicate.

## Results

### Enhanced fitness and growth of the evolved plasmid-carrying bacteria

**Fig. 2:**
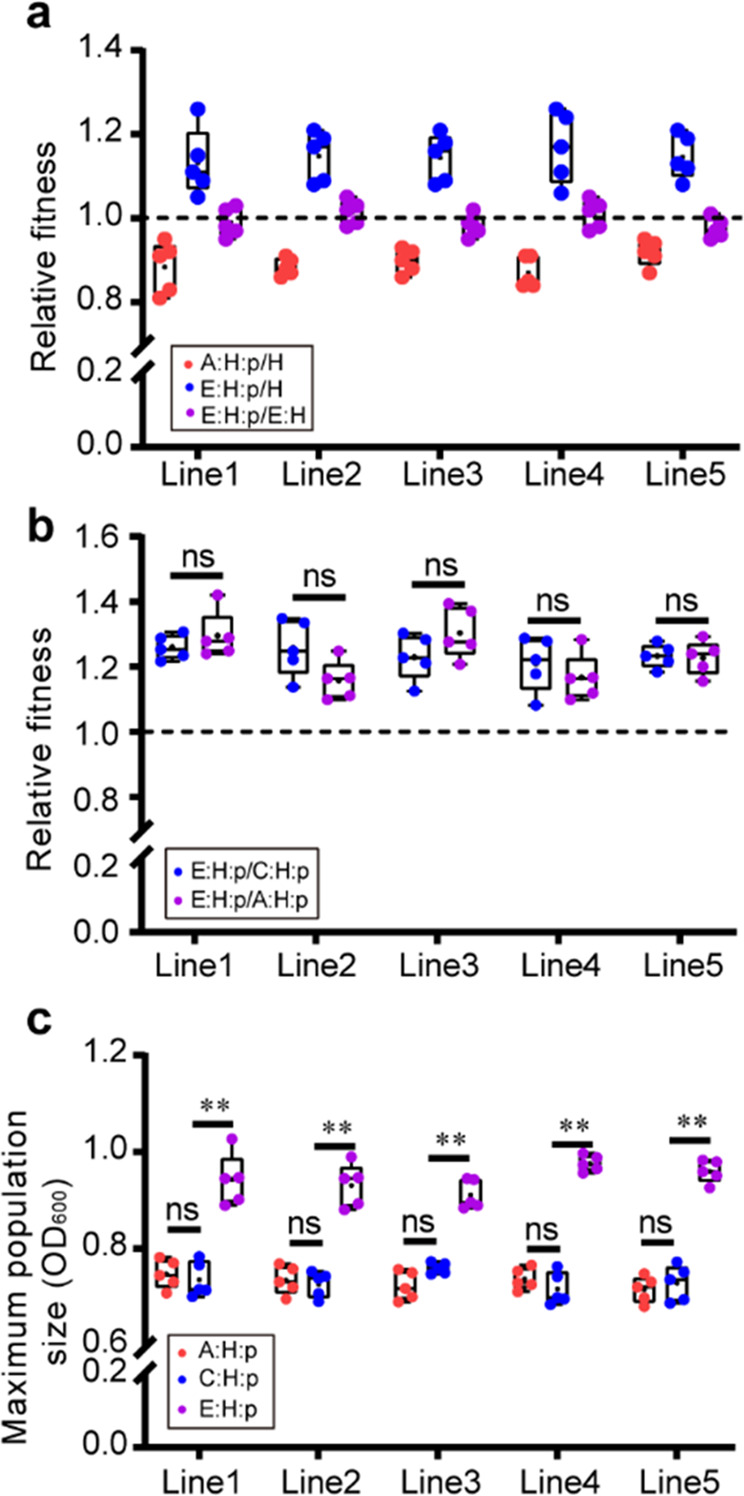
Evolutionary adaptation promoted the fitness and the growth of evolved plasmid-carrying strains. **a** Fitness of plasmid-carrying strains (A:H:p and E:H:p) *versus* isogenic plasmid-free host strains (H) or E:H strains (the plasmid-cured derivatives of E:H:p) in LB medium without antibiotic selection (n = 5). **b** Relative competitive advantages of E:H:p *versus* A:H:p or C:H:p strains *(n* = 5). Statistical comparison was done by independent-sample *t* tests. **c** The maximum population density (OD_600_) reached by bacterial strains after incubation for 24 h (*n* = 5). Statistics by One-Way ANOVA with Tukey post hoc test. Each experiment involved five replicates per evolutionary line, and the five replicates of A:H:p strains per evolutionary line were derived from the same A:H:p strain (*n* = 5). ns no significant difference; *p < 0.05; **p < 0.01.

In the present study, we examined the fitness changes of evolved plasmid-carrying strains in pure culture before looking at their fitness in the mouse gut. The design of the 150–day evolutionary experiments is illustrated in Fig. 1. The results of competition between H and A:H:p strains showed that plasmid carriage imposed a fitness cost on host bacteria, whereas E:H:p strains increased in fitness relative to H strains and exhibited no fitness alterations to E:H strains in antibiotic-free media (Fig. 2a), suggesting no measurable cost of the plasmid after evolution. Additionally, the direct competition between A:H:p and E:H:p strains showed an growth advantage for E:H:p (*t* = 7.47, df = 4, *p* < 0.01; Fig. 2b). These results are consistent with previous studies, in which the fitness cost of RP4 in *E. coli* or *Pseudomonas sp*. is ameliorated after the coevolution underexposure of antibiotics [13, 18, 29]. Moreover, we did not observe plasmid loss in the RP4-carrying *E. coli* strains (A:H:p, C:H:p, and E:H:p), indicating that the plasmid stably persisted in *E. coli*.

To better characterize the growth profiles of evolved strains, we measured bacterial growth curves in terms of OD_600_ and found neither any significant difference in growth rates among A:H:p, C:H:p, and E:H:p strains (ANOVA: F_8,60_ = 1.27, *p* > 0.05; Fig. S1), nor any obvious difference in the maximum population density between A:H:p and C:H:p strains (ANOVA: F_4,40_ = 1.55, *p* > 0.05; Fig. 2c). However, E:H:p strains showed dramatically increased maximum population density compared with A:H:p or C:H:p strains (*p* < 0.01; Fig. 2c), suggesting that long-term antibiotic treatment might increase the maximum population carrying capacity of the evolved strains. Previous studies have demonstrated that indole, as a significant biological–signaling molecule, is involved in regulating the growth of *E. coli* [30, 31]. In this study, we also found that indole production of E:H:p strains was significantly higher than that of A:H:p or C:H:p strains (*p* < 0.01; Fig. S2), as well as the upregulated expression of *tnaA* encoding tryptophanase (*p* < 0.01; Fig. S3). Moreover, the addition of exogenous indole (0.5 mM) to the laboratory culture of A:H:p strains dramatically increased bacterial population density up to the level of E:H:p strains (Fig. S4). Collectively, these results showed that the evolutionary adaptation of plasmid-carrying bacteria to long-term antibiotics could promote the fitness of plasmid-carrying bacteria.

### Genomic mutations and transcriptional modifications associated with bacterial growth phenotypes in the evolved plasmid-carrying bacteria

**Fig. 3:**
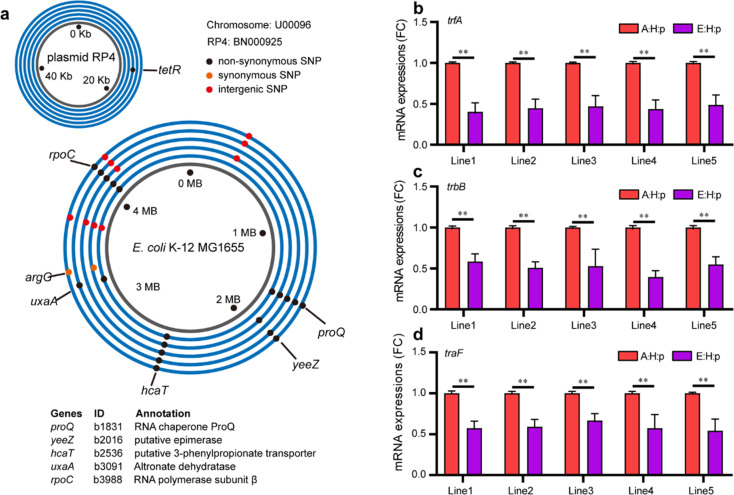
Genomic mutations and transcriptional modifications associated with bacterial growth undergoing long-term antibiotic stress. **a** Mutations observed in E:H:p strains underexposure to long-term antibiotics. Rings represent *E. coli* chromosomes or RP4 plasmid. Dots represent mutations; black dots represent the nonsynonymous SNP sites, orange dots represent synonymous SNP sites and red dots stand for intergenic SNP sites. Blue circles correspond to the genome of experimental lines, with the inner circles corresponding to line 1 and each subsequent circle corresponding to the next line, up to line 5 in the outer blue circles. Detailed information is in Table S4. The mRNA expression of plasmid-borne genes *trbB* (**b**), *traF* (**c**), and *trfA* (**d**) in E:H:p strains; five replicates per evolutionary line, and the five replicates of A:H:p strains per evolutionary line were derived from the same A:H:p strain (n = 5). Statistical comparison was done by Student’s *t* tests. *p < 0.05; **p < 0.01.

Genomic changes, especially accumulations of mutations, are crucial to deciphering how bacteria evolve and adapt at the molecular level [32]. To identify mutations contributing to improved bacterial growth and plasmid fitness, we sequenced the complete genomes of three H strains, five A:H:p strains, five C:H:p strains and five E:H:p strains, all of which were randomly selected from the five evolutionary lines. We observed no chromosome mutations among these isolates of H, A:H:p, and C:H:p strains. It should be noted that mutations were observed in the C:H:p populations (Table S3). In addition, we identified 10 representative mutated chromosomal loci among the five E:H:p strains (Fig. 3a, Table S4). These mutations included one synonymous SNP, three intergenic SNPs and six nonsynonymous SNPs (details in Table S4). Only one mutated site located at gene *tetR* was observed in one of the plasmid genomes (Fig. 3a), suggesting that the enhanced phenotypes of E:H:p strains were associated with the evolution of the host chromosomal genome rather than of the plasmid genome. Furthermore, three nonsynonymous mutations were acquired by multiple evolved isolates, including the gene *proQ* encoding an RNA chaperone that regulates expression of a membrane protein, *hcaT* encoding a transporter that involves uptake of 3-phenylpropionic acid, and *rpoC* encoding a subunit of RNA polymerase. Functional prediction via SnpEff [24] indicated that these altered residues might have moderate effects on protein structures and functions. Moreover, nonsynonymous mutation in the gene altronate dehydratase (*uxaA*) encoding an altronate dehydratase was found in two E:H:p strains, while nonsynonymous mutation in the gene epimerase (*yeeZ*) encoding a putative epimerase was observed in three E:H:p strains. In contrast, we did not observe any similar mutational sites in C:E:H strains compared with E:H:p strains after evolving plasmid-free H strains under antibiotic exposure for 30 days (Table S5).

To study how evolutionary adaptation enhances bacterial fitness, we cured the plasmid RP4 from E:H:p strains and compared the phenotypes of E:H to the ancestral strain H. Results showed that the maximum population density of E:H strains was significantly higher than that of H strains (Fig. S5). Generally, the expression of plasmid-borne genes, especially those involved in plasmid transfer and replication, is considered the major burden for host bacteria. To explore whether such expression influenced alterations to plasmid fitness cost, we determined the mRNA expression of three genes associated with plasmid transfer (*trbB* and *traF*) and replication (*trfA*) in E:H:p strains. The mRNA expression of *trfA*, which encodes the plasmid replication initiation protein, was significantly downregulated in E:H:p strains compared with A:H:p strains (*t* = 38.60, df = 4, *p* < 0.01; Fig. 3b). In addition, mRNA expressions of plasmid transfer related genes *trbB* (t = 15.33, df = 4, *p* < 0.01; Fig. 3c) and *traF* (*t* = 19.46, df = 4, *p* < 0.01; Fig. 3d) were significantly repressed in the E:H:p strains, which was in line with the reduced plasmid conjugative transfer rates (Fig. S6). Collectively, these results demonstrated that genomic mutations and transcriptional modifications jointly participated in the amelioration of plasmid fitness cost.

### Evolutionary adaptation altered the antibiotic resistance of plasmid-carrying bacteria and facilitated the multidrug resistance

**Fig. 4:**
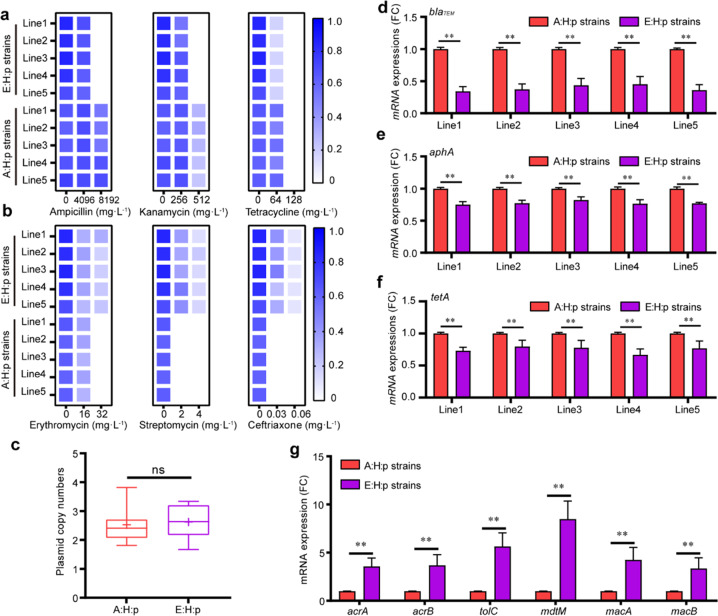
Changes in antibiotic resistance mediated by plasmid-encoded ARGs and chromosome-encoded pumps. MIC of A:H:p and E:H:p strains was revealed by measuring the optical density (OD_600_) of bacterial populations growing in (**a**) a range of concentrations of ampicillin, kanamycin and tetracycline; and (**b**) diverse concentrations of erythromycin, streptomycin and ceftriaxone alone for 24 h (shown in blue); five replicates per evolutionary line (*n* = 5). (**c)** Average RP4 PCN per bacterial cell in A:H:p and E:H:p populations; three replicates per evolutionary line (*n* = 15). The mRNA expression of plasmid-borne ARGs, including *bla*_*TEM*_ (**d**), *aphA* (**e**) and *tetA* (**f**), as quantified by RT-qPCR for A:H:p and E:H:p strains; five replicates per evolutionary line (*n* = 5). (**g**) The mRNA expression of chromosome-encoded pumps (*acrA*, *acrB*, *tolC*, *mdtM*, *macA* and *macB*) as quantified by RT-qPCR for the A:H:p and E:H:p strains, including 25 isolates of E:H:p strains and 5 A:H:p strains (*n* = 5). The five replicates of A:H:p strains per evolutionary line were derived from the same A:H:p strain. Statistical comparison was done by Student’s *t* tests. ns: no significant difference; *p < 0.05; **p < 0.01.

To determine the antibiotic resistance, we tested the MIC of isolates from five evolutionary lines against several antibiotics, including ampicillin, kanamycin, and tetracycline. The RP4 plasmid carries the corresponding antibiotic resistance genes (ARGs) against the three antibiotics mentioned above, respectively *bla*_*TEM*_, *tetA* and *aphA*. We observed no significant difference in resistance to these antibiotics between the A:H:p and C:H:p strains (Fig. S7). However, the MIC of E:H:p strains against ampicillin and kanamycin significantly declined, as shown by a reduction in MIC from >8 to >4 g L^−1^ against ampicillin and from >512 to >256 mg L^−1^ against kanamycin (Fig. 4a). These drops in resistance were not attributed to the change in PCN, as PCN did not differ significantly between A:H:p and E:H:p strains (t = 1.96, df = 4, *p* >0.05; Fig. 4c). Rather, they were associated with reduced transcriptional expression of the plasmid-borne ARGs. For example, expression of *bla*_*TEM*_ and *aphA* was significantly downregulated by 59.2–75.9% (*t* = 27.3, df = 4, *p* < 0.01; Figs. 4d) and 17.5–34.0% (*t* = 19.41, df = 4, *p* < 0.01; Fig. 4e), respectively, according to RT-qPCR results. It should be noted that we observed no obvious decrease in the tetracycline resistance of E:H:p strains *versus* A:H:p or C:H:p strains (Fig. 4a, S7), although expression of *tetA* was significantly downregulated (*t* = 10.98, df = 4, *p* < 0.01; Fig. 4f). On the other hand, the E:H:p strains showed increased resistance against erythromycin, streptomycin and ceftriaxone (Fig. 4b) and elevated mRNA expression of chromosome-encoded efflux pumps, including acriflavine resistance proteins A (*acrA*: *t* = 11.47, df = 28, *p* < 0.01; Fig. 4g) and B (*acrB*: *t* = 9.24, df = 28, *p* < 0.01; Fig. 4g), outer-membrane protein (*tolC*: *t* = 12.77, df = 28, *p* < 0.01; Fig. 4g), multidrug-resistance protein (*mdtM*: *t* = 15.52, df = 28, *p* < 0.01; Fig. 4g), and macrolide export proteins A (*macA*: *t* = 9.76, df = 28, *p* < 0.01; Fig. 4g) and B (*macB*: *t* = 8.28, df = 28, *p* < 0.01; Fig. 4g).

### Evolutionary adaptation increased the biofilm-forming capacity of plasmid-carrying bacteria

**Fig. 5:**
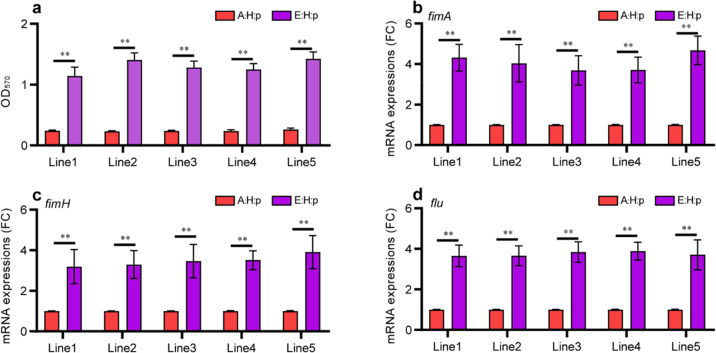
Evolutionary adaptation increased the biofilm-forming capacity of plasmid-carrying bacteria. **a** Biofilm production (OD_570_) of A:H:p and E:H:p strains as analyzed by 96-well plate assay; five replicates per evolutionary line (*n* = 5). The mRNA expression of biofilm formation–related genes *fimA* (**b**), *fimH* (**c**) and *flu* (**d**) in E:H:p strains; five replicates per evolutionary line (*n* = 5), and the five replicates of A:H:p strains per evolutionary line were derived from the same A:H:p strain. Statistical comparison was done by Student’s *t* tests. *p < 0.05; **p < 0.01.

A previous study suggested that an increase in bacterial population size in liquid culture may be associated with increased biofilm formation [33]. Bacterial biofilm is also recognized as a crucial factor influencing bacterial antibiotic resistance, growth, and adaption in novel niches [34]. In the present study, we investigated the biofilm yield of A:H:p, C:H:p, and E:H:p strains via crystal violet staining (monitoring OD_570_). We observed no significant difference in the biofilm yield between A:H:p and C:H:p strains (*t* = 1.49, df = 4, *p* > 0.05; Fig. S8). However, the biofilm yield of E:H:p strains was increased by four- to six-fold over that of A:H:p strains (*t* = 21.08, df = 4, *p* < 0.01; Fig. 5a). Considering that the biofilm-forming capability mainly depends on bacterial adhesion [35, 36], we performed RT-qPCR to explore the expression of type 1 fimbrial-protein pilus genes (*fimA* and *fimH*) and the surface adhesin gene (*flu*), which are involved in bacterial adhesion [37, 38]. The mRNA expression of *fimA* (*t* = 16.45, df = 4, *p* < 0.01; Fig. 5b) and *fimH* (*t* = 19.99, df = 4, *p* < 0.01; Fig. 5c) was considerably increased in E:H:p strains *versus* A:H:p strains. The mRNA expression of *flu*, which encodes the major outer-membrane protein antigen 43 (Ag43), was also significantly upregulated in E:H:p strains (*t* = 55.71, df = 4, *p* < 0.01; Fig. 5d), thereby likely promoting cell-to-cell adhesion and biofilm maturation [39, 40]. Collectively, these results suggested that evolutionary adaptation under long-term antibiotic exposure enabled the plasmid-carrying strains to develop an enhanced biofilm-forming capacity.

### In vivo colonization, transfer, and survival of exogenous plasmid-carrying bacteria in mice

**Fig. 6:**
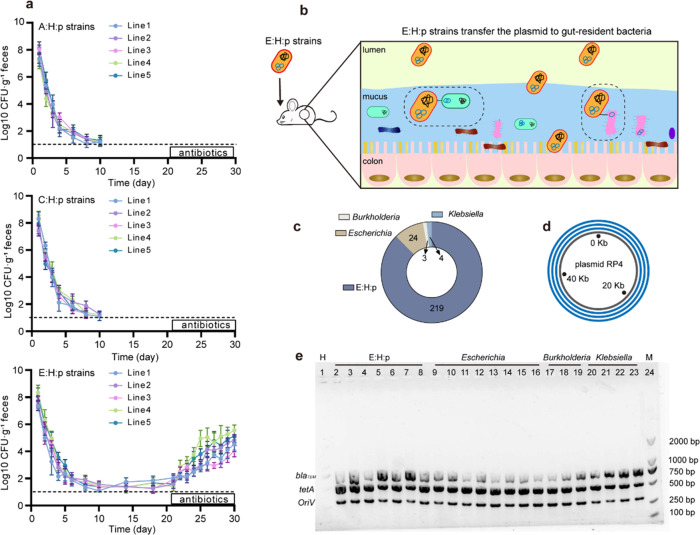
Gut colonization by evolved plasmid-carrying bacteria, and plasmid transfer to gut-resident bacteria. **a** Parallel assays of murine-gut colonization by five lines of A:H:p, C:H:p and E:H:p strains, including E:H:p strains and other RP4-carrying gut-resident bacteria in fecal samples. **b** Diagram of E:H:p strains transferring RP4 plasmid to gut-resident bacteria. **c** Plasmid RP4 recipients isolated on the last day of the colonization test were identified by Sanger sequencing of the 16S rRNA gene, including E:H:p strains, gut-resident *Escherichia* (*E. coli*, *E. fergusonii*)*, Klebsiella* (*K. pneumonia*, *K. singaporensis*) and *Burkholderia* (*B. fungorum*). **d** No mutations were observed on RP4 plasmid from these randomly selected gut-resident bacteria. **e** Identification of RP4 plasmid in bacterial isolates from murine fecal samples by PCR.

The invasion of outside bacteria into a host occurs frequently, yet colonization by the invading bacteria does not usually happen, this is because of host defense mechanisms and colonization resistance endowed by endogenous microbiota [41, 42]. To explore whether evolutionary adaptation influenced bacterial colonization, this study compared the colonization for the E:H:p, C:H:p, and A:H:p strains in mice. After each mice was intragastrically administered with 2 × 10^9^ CFU, cell counts of the A:H:p and C:H:p strains dropped quickly, and both strains were finally eliminated (below the limit of detection, 10 CFU g^−1^ feces) after 10 days (Fig. 6a). By contrast, the E:H:p strains persisted at ~10^2^ CFU g^−1^ feces even after 20 days, indicating a better survival for E:H:p strains in mice. More importantly, subsequent administration of antibiotics significantly boosted the E:H:p proliferation from ~10^2^ CFU g^−1^ feces on day 20 to ~10^4^ CFU g^−1^ feces on day 30 (Fig. 6a). In addition, no additional new mutations were observed on the chromosome or plasmid genome of E:H:p strains during the process of colonization, by sequencing five isolates of E:H:p strains from murine fecal samples after 30 days of exposure. These results collectively demonstrated that evolutionary adaptation of plasmid-carrying bacteria to long-term antibiotic exposure could prolong their survival period in murine gastrointestinal tract.

We further tested whether the RP4 plasmid could transfer horizontally from the donor (E:H:p strains) to endogenous bacteria by isolating and verifying RP4-carrying strains from mouse feces. Indeed, the plasmid RP4 carried by the evolved strains was transferred to other murine gut-resident bacteria, including *Escherichia*, *Klebsiella,* and *Burkholderia* (Fig. 6b–c; Table S6). WGS results showed that no mutational sites occurred on plasmid DNA from these RP4-carrying gut-resident bacteria in comparation with E:H:p strains (Fig. 6d), and the prevalence of RP4 was confirmed by the PCR and agarose gel electrophoresis (Fig. 6e). In conclusion, the survival time of exogenous plasmid-carrying strains (E:H:p strains) in gut microbiota was reasonably extended by phenotypic and genotypic modifications during evolutionary adaptation. The horizontal transfer of plasmid in the murine gut was likely facilitated by better survival of evolved strains induced by evolutionary adaptation to antibiotic exposure.

To determine the survival ability of E:H:p strains at systemic sites, the bacteria were administered to mice via intraperitoneal injection. The competitive index of E:H:p strains in murine livers and spleens were significantly higher than that of A:H:p or C:H:p strains without antibiotic exposure (*p* < 0.01; Fig. S9), indicating their increased survival in mice.

## Discussion

### Phenotypic and genotypic modifications were beneficial to the survival of the evolved plasmid-carrying bacteria in gut microbiota

Understanding the colonization ability of evolved plasmid-carrying bacteria has significant ecological and health implications. Previous studies mainly focused on the evolutionary dynamics and fates of plasmid-carrying bacteria by using in vitro evolutionary models [18, 43–45]. However, very few studies have revealed whether the plasmid-carrying bacteria evolved in the presence of antibiotics, have an enhanced ability to colonize the mammalian gut, which represents one of the greatest transmission pathways of bacteria and plasmids from the environment into mammals.

This study combined a long-term in vitro evolutionary model with in vivo colonization experiments in mice to investigate colonization of the murine intestinal niche by evolved plasmid-carrying bacteria. We found that the evolved strains acquired an enhanced ability to prolong its survival period in murine gut microbiota even without antibiotics, but antibiotic regimens greatly increased the likelihood that these strains would colonize the gut. In addition, the plasmids were transferred into endogenous bacteria such as *Escherichia*, *Klebsiella* and *Burkholderia*. Conversely, the ancestral strains (A:H:p) and the control strains (C:H:p) could not colonize the murine gut, regardless of antibiotic stress.

To explore how the evolved strains acquired this enhanced colonization ability, we dissected the underlying mechanism by trying to link the genomic mutations and transcriptional modification to phenotypic alterations of the evolved strains. The allelic reconstruction of the three reoccurring mutations alone was not related to the changes of bacterial phenotypes (Fig. S10). Even so, we might not be able to rule out that the three mutations (*proQ*, *hcaT,* and *rpoC*) in combination participated in regulating bacterial phenotypes, because we observed the significant phenotypic alterations of enhanced bacterial growth in EH strains (plasmid-cured strains) compared to H strains (Fig. S5). Indeed, mutations to gene *rpoC* were reported to confer antibiotic resistance and fast growth rate in bacteria [46, 47]. Another previous study also confirmed that *rpoC*, which encodes the DNA-directed RNA polymerase subunit β’ to regulate the transcription of DNA into RNA, is involved bacterial phenotypic changes [48]. The gene *proQ* was involved in regulating bacterial motility and pathogenesis in *vivo* [49]. And the gene *hcaT* encodes a putative 3-hydroxypropionic acid transporter in *E. coli* K-12 MG1655, which supports the catabolism of different phenylpropanoid compounds [50]. Further studies are needed to dissect the role of these mutations in altered phenotypes and transcriptional modifications. Additionally, transcriptional modifications were also partly responsible for the altered phenotypes of evolved strains, evidenced from the downregulation of plasmid-borne genes and the upregulated expression of *tnaA*.

The mechanistic explanations for the evolutionary adaptation of plasmid-carrying bacteria (e.g. RP4 and RK2) linked many genomic mutations to plasmid cost and the sensitivity to antibiotics [13, 18, 29]. For example, chromosomal mutations on accessory helicases genes such as *Xpd*/*Rad3* and *UvrD* could improve the DNA or RNA metabolism of RP4-carrying *Pseudomonas sp*. via interacting with DNA helicase DnaB or plasmid replication protein TrfA; and mutations on β-subunit of RNA polymerase gene *RpoB* ameliorate plasmid cost through the mutated helicases [13]. In addition, the mutation in *ompF* (outer-membrane porin) and *acrR* (encoding AcrAB operon repressor) could increase the tetracycline resistance of RK2-carrying *E. coli* strains, while mutations in *ychH* (stress response gene) and *adhE* (aldehyde-alcohol dehydrogenase) contribute to improve bacterial growth [18, 29]. Although the mutated genes revealed in this study are different with previous studies, our findings could be well complemented to the mechanistic explanation for the evolutionary adaptation of plasmid-carrying bacteria with particular for IncP-1 plasmids.

In this study, the influence of growth medium in phenotypic alterations (such as plasmid fitness cost) was ruled out for evolved strains. The indole component has been revealed as a signaling molecule regulating bacterial growth and colonization [30, 51–54]. The positive effect of indole on bacterial growth was also confirmed by testing indole contents and assaying bacterial growth promoted by the addition of indole. Indole was also likely involved in multidrug resistance of evolved strains because the addition of indole transcriptionally upregulated the expression of chromosomal efflux pump genes (*acrA*, *acrB*, and *mdtM*) (Fig. S11). Indeed, the acrA/B–tolC and mdtM efflux pumps can confer tetracycline resistance [55, 56], thereby compensating for the decrease in the expression of *tetA*. In the evolved plasmid-carrying bacteria, the ampicillin and kanamycin resistance conferred by plasmid RP4 was dramatically reduced during the evolutionary process due to the decreased RNA transcripts of the plasmid-borne *bla*_*TEM*_ and *aphA* genes in the evolved strains. However, the efficiency of these pumps was likely not sufficient to compensate for the decrease in plasmid-encoded resistance to ampicillin and kanamycin in E:H:p strains, given that MIC level was as high as >4 g·L^−1^ for ampicillin and >250 mg·L^−1^ for kanamycin.

As evolved strains exhibited higher population size at the stationary phase of bacterial growth, they were likely more tolerant to toxic products of catabolism and the exhaustion of nutrients [57], which might benefit evolved strains surviving in complicated intestinal environments. Increased bacterial biofilm might have facilitated the evolved strains into the mucus layer of the murine intestine tract [58]. In addition, the evolved strains can generate more indole production, which might be favorable to promote the growth of evolved strains, and also affect the proliferation of resident intestinal microflora [59]. Collectively, these phenotypic alterations and genotypic modifications likely promoted the evolved strains to compete in mice, thus contributing to their survival and subsequent colonization in murine gut microbiota.

### Implications for plasmid-carrying bacterial colonization facilitated by evolutionary adaptation to antibiotic exposure

Recently, the spread of multidrug pathogens has raised worldwide concerns, particularly in terms of levels of antibiotics being introduced from environmental settings (e.g., aquaculture and livestock farms [60, 61], and wastewater treatment plants [62, 63]). Herein, our findings showed that long-term antibiotic exposure facilitated plasmid-carrying bacterial colonization of gut microbiota. These results also highlighted the transmission risk of plasmid-borne ARGs from the environment toward mammals. Indeed, previous studies have documented various shared commensal bacteria in the environment–animal and animal–human confluences [64–66]. For example, swine farm exposure was found to shape the gut microbiomes and resistomes of students with 3 months’ occupational exposure, resulting in enrichment of potentially pathogenic taxa and AMR genes [64]. However, these previous studies did not explain how this has been happening. Our results might provide relevant evidence that plasmid-carrying strains tend to acquire prolonged survival ability via evolutionary adaptation to antibiotic exposure, thereby enabling the evolved strains to colonize a novel niche (e.g., the murine gut, as in this study). Alarmingly, antibiotic resistance could be spread by the transfer of plasmids among gut-resident bacteria.

Our results demonstrated that evolutionary adaptation of plasmid-carrying strains to antibiotic exposure poses the risk of promoting the spread of resistance plasmids from the environment towards mammals. These findings raise an alarm that bacteria and plasmids exclusive to the environment are quietly evolving the ability to invade mammals, which calls the need to fight against AMR in a One Health perspective [67].

## Supplementary information

Supporting information

## Data Availability

Bacterial-genome sequence data are available at the US National Center for Biotechnology Information (NCBI; Bethesda, MD, USA; Accession No. PRJNA671781).
