# Supplementary material for: Colonization of gut microbiota by plasmid-carrying bacteria is facilitated by evolutionary adaptation to antibiotic treatment
Source: ISME J. 2021 Dec 13;16(5):1284–93. doi: 10.1038/s41396-021-01171-x (PMC9038720; doi:10.1038/s41396-021-01171-x)
Supplement: Supplementary file 1 — Supporting information [file 41396_2021_1171_MOESM1_ESM.docx]

# Supplementary information

# Colonization of gut microbiota by plasmid-carrying bacteria is facilitated by evolutionary adaptation to antibiotic treatment

## Running title: Colonization of gut microbiota by plasmid-carrying bacteria

Peng Zhang ^a, b^ , Daqing Mao ^c,^* , Huihui Gao ^c^, Liyang Zheng ^c^, Zeyou Chen ^a^, Yuting Gao ^a^, Yitao Duan ^a^, Jianhua Guo ^d,^* , Yi Luo ^b,^ ^a,^* and Hongqiang Ren ^b^

^a^ College of Environmental Sciences and Engineering, Nankai University, Tianjin 300350, China

^b^ State Key Laboratory of Pollution Control and Resource Reuse, School of the Environment, Nanjing University, Nanjing 210046, China

^c^ School of Medicine, Nankai University, Tianjin 300071, China

^d^ Australian Centre for Water and Environmental Biotechnology, The University of Queensland, St. Lucia, Brisbane, QLD 4072, Australia

*Address corresponding to Daqing Mao, E-mail address: maodq@nankai.edu.cn;

Jianhua Guo, E-mail address: jianhua.guo@uq.edu.au;

Yi Luo, E-mail address: [luoy@nankai.edu.cn](mailto:luoy@nankai.edu.cn).

The supplementary information includes three appendices. The first appendix contains information about additional methods and protocols. The second appendix provides details on additional figures. The third appendix describes supporting results in tables.

**I. Supplemental Methods**

**II. Supplemental Figures**

**III. Supplemental Tables**

## I. Supplemental Methods

### Text S1. Growth curves

The bacterial growth curves were monitored by a Bioscreen C Microbiological Growth Analyser (Labsystems, Helsinki, Finland). Specifically, each well of the honeycomb microplates loading with 198 µL fresh LB broth with or without antibiotics, were inoculated with 2 µL tested strains (H, A:H:p, C:H:p and E:H:p strains, 5×10^8^ CFU·mL^-1^), and cultured with the shaking mode at 37 °C, and the bacterial concentration of OD600 was recorded every 15 min over a 20 h period. *Growthcurver* package in R was used to analyze bacterial growth curves and obtain interpretable metrics [1], including the intrinsic growth rate (r) of the population that would occur if there were no restrictions imposed on total population size.

### Text S2. Plasmid copy number determination

To determine the plasmid copy number, we performed droplet digital PCR (ddPCR) on the Naica Crystal Digital ^TM^ PCR System (Stilla Technologies, Villejuif, France) with Sapphire chips (Stilla Technologies, Villejuif, France).The template DNA (2 μl per replicate) was assembled in 25 μl PCR mixtures using 1 × PerfeCTa qPCR ToughMix, UNG (Quanta Biosciences, Gaithersburg, MD, USA), 1 ×Alexa Flour 647 reference Fluorescent dye, 1 ×EVAGREEN, 1 μl of each primer (*dxs* and *traJ*, 10 μM). PCR consisted of 5 min at 95 °C, followed by 45 cycles of 95 °C for 15 s and 60 °C for 30 s. The data were captured by the software of Crystal Reader in digital PCR data captured equipment Naica Prism3 (Stilla Technologies, France).

### Text S3. Hydroxylamine-based indole assay to determine indole released from bacteria

The concentration of indole released by bacteria was tested with previously described hydroxylamine-based methods [2]. Briefly, 100 µL indole standard solution (prepared in 70% ethanol solution at concentrations of 50 to 500 µM) or bacterial secretions from in vitro or in vivo experiments was inoculated with 25 µL of 5.3 M NaOH solution (Solarbio, Beijing, CN) and 50 µL of 0.3 M hydroxylamine hydrochloride (Macklin, Shanghai, CN) for 15 min at room temperature. Then, 125 µL of 2.7 M H_2_SO_4_ was added, mixed thoroughly, and hold statically at room temperature for at least 30 min. The pink solution was recorded spectrophotometrically by plates reader at 530 nm for measurement of indole content. The bacterial secretion samples from in vitro incubation were prepared by centrifugation 1 mL of bacterial culture in LB at 15000 rpm for 15 min at room temperature, and 100 µL of the supernatant was used to test indole content as described above. As for murine fecal samples from in vivo bacterial gut-colonization experiment, 250 mg of stool was suspended in 750 µL 70% ethanol solution, and then the samples were mixed thoroughly and incubated at 70 °C for 10 min. Subsequently, the mixtures were vortexed thoroughly and centrifuged at 15,000 rpm for 20 min at room temperature to separate bacterial secretion. Finally, 100 µL stool supernatants were used to examine indole production.

### Text S4. Biofilm formation determination

The biofilm formation properties of H, A:H:p and E:H:p strains were determined by 96-well plate assay as described previously [3]. Briefly, H, A:H:p and E:H:p strains were grown for 12 h and appropriately diluted (5×10^8^ CFU·mL^-1^), 2 μL of each dilutions was pipetted into 5 wells in fresh 96-well plates containing 198 μL LB broth, then the plates were incubated at 37 ℃ for 48 h. Afterwards, the plates were washed with water for 2 times to remove planktonic bacteria, the biofilm was stained with 0.1% crystal violet solution (125 μL per well) for ten min, and then the plates were washed with water for 2 times to remove any crystal violet that is not specifically staining the adherent bacteria. After the air-dry of the plates for 30 min, 30% acetic acid (200 μL per well) were used to solubilize the biofilms, and the plates was recorded by plates reader (Tecan F50) at a wavelength of 570 nm.

### Text S5. Plasmid conjugative transfer assays

Conjugation assays between donor and recipient bacteria were conducted in antibiotic-free media to characterize the plasmid conjugative rates. Ancestral strains or evolved strains are donors, and the recipients include *E. coli* J53 resistant to sodium azide and *E. coli* HB101 resistant to streptomycin. And the detailed information about the related method were according to previous report [4].

### Text S6. Curing RP4 from evolved strains

The plasmid was cured from evolved strains using the pCURE curing system [5]. The genes on RP4 such as *oriV*, *parD*, *korA* and *incC*, were ligated into the pk18 streptomycin-resistant vector that contains the selective marker gene *sacB*. The resultant plasmids were introduced into electrocompetent E:H:p strains and selected with 25 μg·mL^-1^ streptomycin, then single colony was streaked on LB plates with 25 μg·mL^-1^ streptomycin and plates with 25 μg·mL^-1^ streptomycin + 5% sucrose. These sucrose-sensitive isolates were tested by PCR for the presence of the curing plasmid (gene *sacB*) and absence of RP4 plasmid (gene *bla and tetA*). To obtain segregation of the curing plasmid, successfully cured isolates were cultured in antibiotic-free LB medium for 24h, and sergeants were selected on antibiotic-free LB plates with 5% sucrose. Sensitivity to antibiotics (ampicillin, tetracycline, and streptomycin) and PCR (gene *sacB*, *bla*, and *tetA*) were used to confirm the loss of plasmid RP4 and pK18.

### Text S7. Single-base editing in WT *E. coli* strains

To explore the role of mutational genes in bacterial phenotypes, we performed allelic reconstructions for individual SNPs (*proQ*, *hcaT* and *rpoC*) with CRISPR/Cas9 systems as previously described [6, 7]. In brief, the pCas9 plasmid was electroporated in *E. coli* K-12 MG1655 strains. Electroporation was performed at 200 Ω and 2.5 kV, and a 0.2-cm electroporation cuvette was used. Thereafter, bacterial cells were immediately transferred to 900 mL of SOC and incubated at 30°C, at 180 rpm for 1 h, and then spread on LB agar containing D-galactose (0.5%) and chloramphenicol (25 μg/mL) and incubated for 24 h at 30°C. Surviving cells were enumerated to confirm the presence of pCas9. Then, the pKD46 plasmid was electroporated in *E. coli* strains harboring pCas9, and the *E. coli* strains harboring pCas9 and pKD46 were selected with LB agar containing D-galactose (0.5%), chloramphenicol (25 μg·mL^-1^), and ampicillin (50 μg·mL^-1^). The corresponding donor DNA and ptargetF plasmid containing sgRNA (*rpoC*-F: gcgaacccccggtactgctg, *rpoC*-R: cagcagtaccgggggttcgc; *proQ*-F: gcaacgcgtgtcgttcttga, *proQ*-R: tcaagaacgacacgcgttgc; *hcat*-F: cccataatgcccctgcgcac,

*hcat*-R: gtgcgcaggggcattatggg) of mutational sites (*proQ_1915290_*, *hcaT_2667006_*, and *rpoC_4186605_*) were co-electroporated in *E. coli* strains harboring pCas9 and pKD46 plasmid, and screened on LB agar plates containing kanamycin (25 μg·mL^-1^). After 20 h at 37°C, 20 colonies (per electroporation) were randomly selected, and Sanger sequencing was performed to confirm the desired base editing in the bacterial genome using primers (Table S2). And five base-editing clones for each mutational genes were selected for further phenotypic analysis.

## II. Supplemental Figures


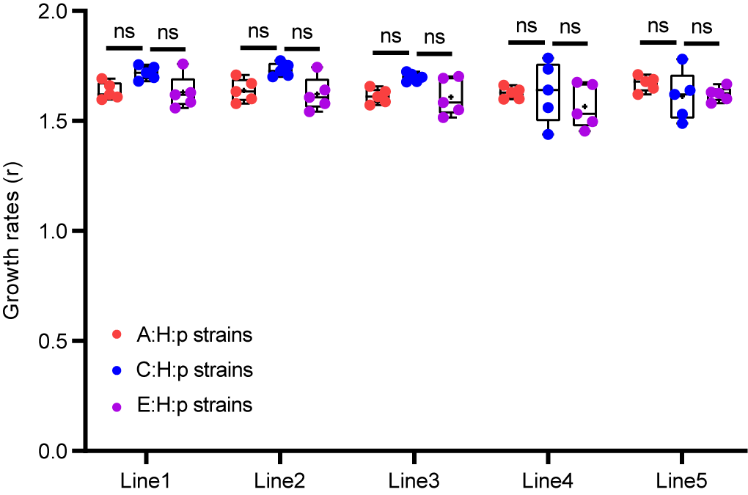


### Figure S1. The growth rates (r) of A:H:p, C:H:p and E:H:p. The intrinsic growth rates (r) that would occur if there were no restrictions imposed on total population size, was obtained by the R package (*Growthcurver*) from the growth curves of these strains, including 5 replicates in each evolutionary line, and the five replicates of A:H:p strains per evolutionary line were derived from the same A:H:p strain. Statistics by One-Way ANOVA with Tukey post-hoc test. ns: no significant difference.


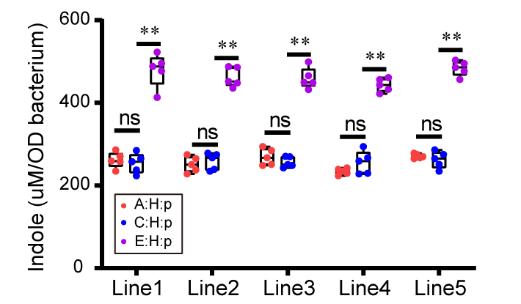


### Figure S2. The indole production of A:H:p, C:H:p and E:H:p strains from each lines. The indole concentration was normalized to the value of OD_600_ of each strain, including 5 replicates in each evolutionary line, and the five replicates of A:H:p strains per evolutionary line were derived from the same A:H:p strain. Statistics by One-Way ANOVA with Tukey post-hoc test. ***p* < 0.01; ns: no significant difference.

###
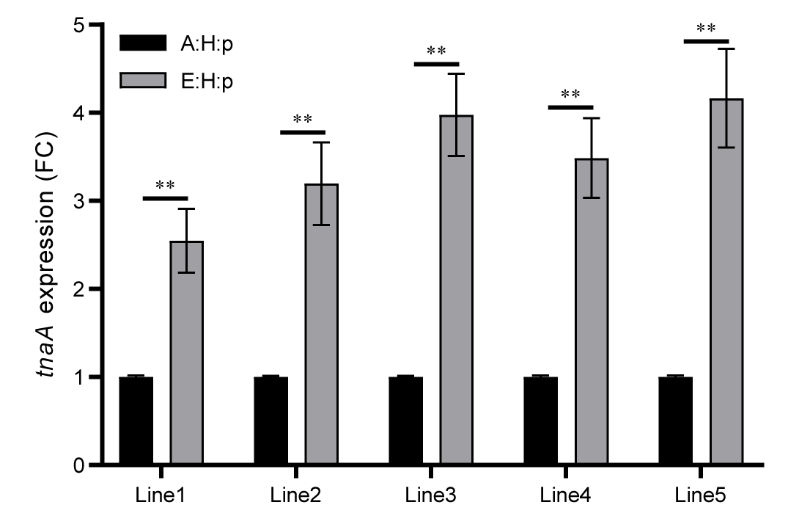


### Figure S3. The mRNA expression of gene *tnaA* encoding tryptophanase, as quantified by RT-qPCR for A:H:p and E:H:p strains; five replicates per evolutionary line, and the five replicates of A:H:p strains per evolutionary line were derived from the same A:H:p strain. Statistics by Student’s *t* test. ***p* < 0.01.


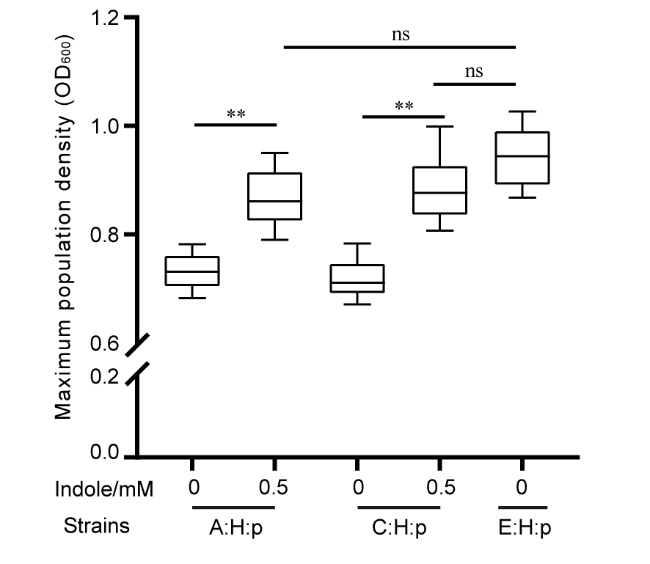


### Figure S4. Indole addition enhanced the maximum population density of A:H:p strains. The average maximum population density (OD_600_) of A:H:p, C:H:p and E:H:p strains without indole addition, A:H:p and C:H:p strains with 0.5 mM of indole addition, including 25 replicates of A:H:p, C:H:p and E:H:p strains, and the five replicates of A:H:p strains per evolutionary line were derived from the same A:H:p strain. Statistics by One-Way ANOVA with Tukey post-hoc test. ***p* < 0.01; ns: no significant difference.

###
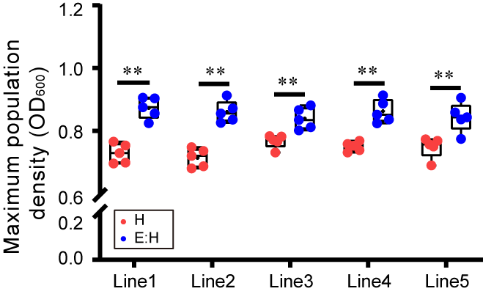


### Figure S5. The maximum population density (OD_600_) of H and E:H strains (the plasmid-cured derivative of E:H:p). Statistics by Student’s *t* test. ***p* < 0.01.


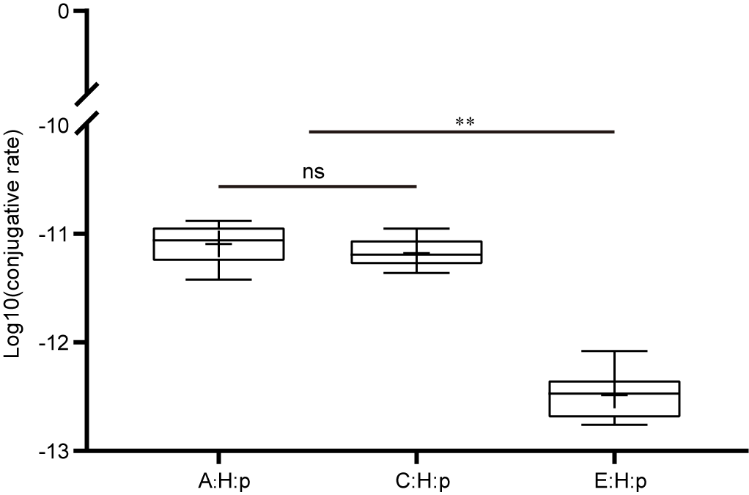


### Figure S6. The plasmid conjugative transfer rates for A:H:p, C:H:p, and E:H:p strains. Five replicates of A:H:p strains and 25 replicates of C:H:p and E:H:p strains were involved in the test. Statistics by One-Way ANOVA with Tukey post-hoc test. ***p* < 0.01; ns: no significant difference.


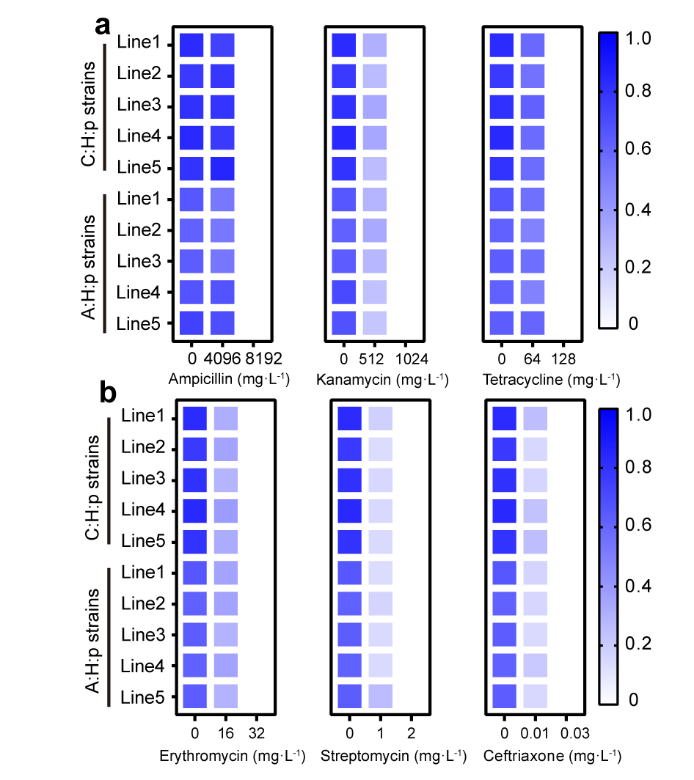


### Figure S7. The profiles of antibiotic resistance in A:H:p and C:H:p strains. The MIC of A:H:p and C:H:p strains revealed by measuring the optical density (OD_600_) of bacterial populations growing in (a) a range of concentrations of ampicillin, kanamycin and tetracycline, and (b) diverse concentration of erythromycin, streptomycin and ceftriaxone alone for 24 h, that is blue-coded as indicated in the legend, including 5 replicates in each evolutionary line, and the five replicates of A:H:p strains per evolutionary line were derived from the same A:H:p strain.


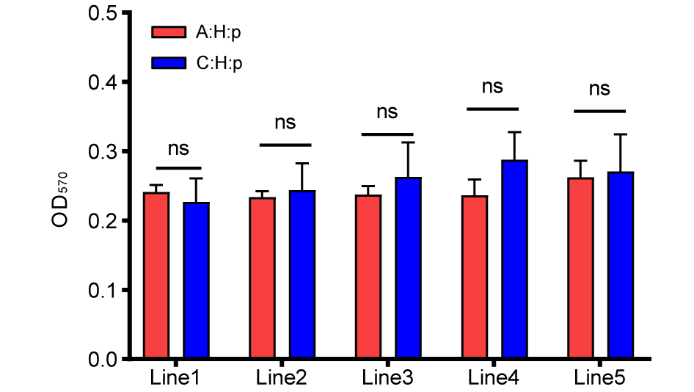


### Figure S8. The biofilm-forming capacity of A:H:p and C:H:p strains. Biofilm production of A:H:p and C:H:p strains was analyzed by 96-well plate assay, including 5 replicates in each evolutionary line, and the five replicates of A:H:p strains per evolutionary line were derived from the same A:H:p strain. Statistics by Student’s *t* test. ns: no significant difference.

###
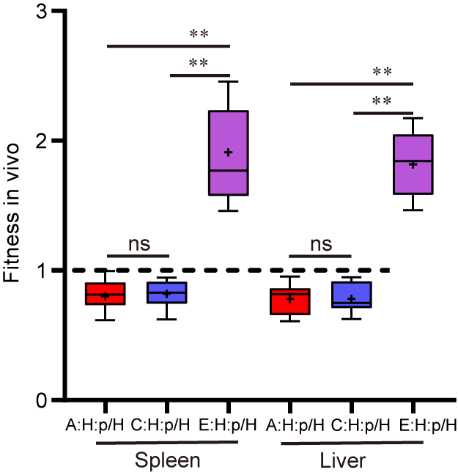


### Figure S9. The competitive index of A:H:p, C:H:p and E:H:p versus parental H strains in murine liver and spleen without antibiotic exposure, including 25 E:H:p strains, 25 C:H:p strains and 5 A:H:p strains, each mice was administered with one bacterial strain. Statistics by One-Way ANOVA with Tukey post-hoc test. ***p* < 0.01; ns: no significant difference.

###
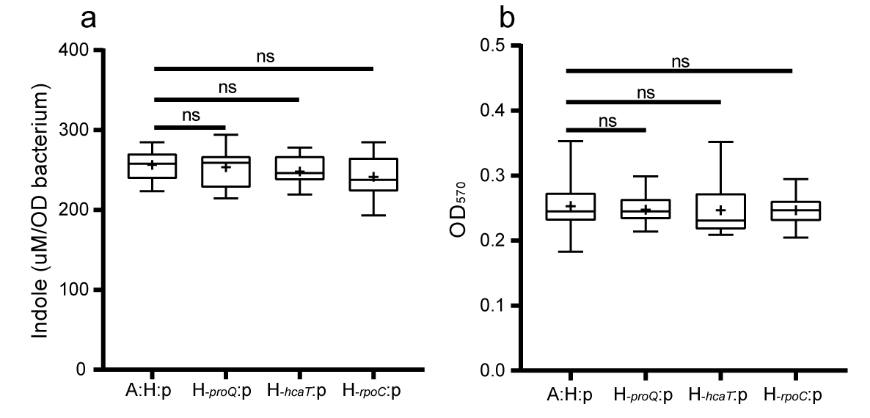


### Figure S10. Indole yield (a) and biofilm production (b) of A:H:p and allelic reconstructed strains with individual SNPs (*proQ*, *hcaT* and *rpoC*). Each experiment involved five replicates of A:H:p strains and five allelic reconstructed strains. Statistics by One-Way ANOVA with Tukey post-hoc test. ns: no significant difference.

###
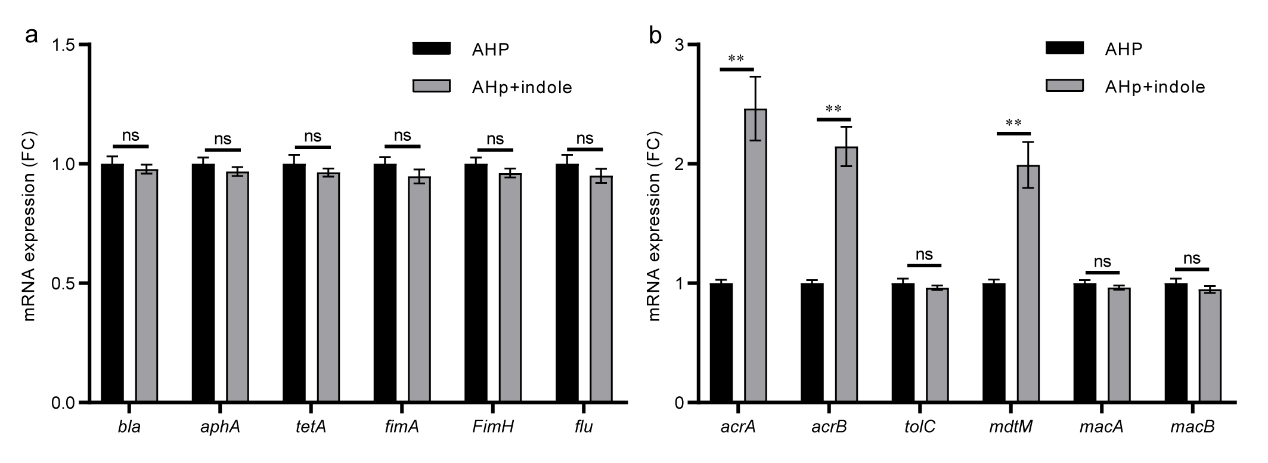


### Figure S11. The influence of indole addition on the RNA expression of gene involved in plasmid-borne ARGs, biofilm-formation, and efflux pumps. The mRNA expression of (a) plasmid-borne ARGs (*bla*, *aphA*, and *tetA*) and biofilm-formation genes (*fimA*, *fimH*, and *flu*), and (b) efflux pumps genes (*acrA*, *acrB*, *tolC*, *mdtM*, *macA*, and *macB*), as quantified by RT-qPCR for A:H:p and A:H:p strains with indole (0.5 mM); five A:H:p strains were chosen to be used in the test. Statistics by Student’s t test. ***p* < 0.01; ns: no significant difference.

## III. Supplemental Tables

### Table S1. The strains used in the experiment

| Number | Clones | Treatments | Plasmids | MIC (μg·mL^-1^) | | | | | |
| --- | --- | --- | --- | --- | --- | --- | --- | --- | --- |
|  |  |  |  | ampicillin | Kanamycin | Tetracycline | Erythromycin | Streptomycin | Ceftriaxone |
| 1 | Plasmid-free *E. coli* K-12 MG1655 was treated as the parental **H**ost:  H-1 | 0 day | n | >4 | >4 | >2 | >16 | >1 | 0.01 |
| 2 | H-2 | 0 day | n | >4 | >4 | >2 | >16 | >1 | 0.01 |
| 3 | H-3 | 0 day | n | >4 | >4 | >2 | >16 | >1 | 0.01 |
| 4 | H-4 | 0 day | n | >4 | >4 | >2 | >16 | >1 | 0.01 |
| 5 | H-5 | 0 day | n | >4 | >4 | >2 | >16 | >1 | 0.01 |
| 6 | An original RP4-carrying *E. coli* K12 MG1655 strain was treated as the **A**ncestral **H**ost with **p**lasmid: A:H:p-1 | 0 day | RP4 | >8192 | >512 | >64 | >16 | >1 | 0.01 |
| 7 | A:H:p-2 | 0 day | RP4 | >8192 | >512 | >64 | >16 | >1 | 0.01 |
| 8 | A:H:p-3 | 0 day | RP4 | >8192 | >512 | >64 | >16 | >1 | 0.01 |
| 9 | A:H:p-4 | 0 day | RP4 | >8192 | >512 | >64 | >16 | >1 | 0.01 |
| 10 | A:H:p-5 | 0 day | RP4 | >8192 | >512 | >64 | >16 | >1 | 0.01 |
| 11 | D25-E:H:p-1 | A+K  (25 days) | RP4 | >8192 | >512 | >64 | ND | ND | ND |
| 12 | D25-E:H:p-2 | A+K  (25 days) | RP4 | >8192 | >512 | >64 | ND | ND | ND |
| 13 | D25-E:H:p-3 | A+K  (25 days) | RP4 | >8192 | >512 | >64 | ND | ND | ND |
| 14 | D25-E:H:p-4 | A+K  (25 days) | RP4 | >8192 | >512 | >64 | ND | ND | ND |
| 15 | D25-E:H:p-5 | A+K  (25 days) | RP4 | >8192 | >512 | >64 | ND | ND | ND |
| 16 | D50-E:H:p-1 | A+K  (50 days) | RP4 | >8192 | >512 | >64 | ND | ND | ND |
| 17 | D50-E:H:p-2 | A+K  (50 days) | RP4 | >4096 | >256 | >64 | ND | ND | ND |
| 18 | D50-E:H:p-3 | A+K  (50 days) | RP4 | >8192 | >512 | >64 | ND | ND | ND |
| 19 | D50-E:H:p-4 | A+K  (50 days) | RP4 | >8192 | >512 | >64 | ND | ND | ND |
| 20 | D50-E:H:p-5 | A+K  (50 days) | RP4 | >8192 | >512 | >64 | ND | ND | ND |
| 21 | D100-E:H:p-1 | A+K  (100 days) | RP4 | >4096 | >256 | >64 | ND | ND | ND |
| 22 | D100-E:H:p-2 | A+K  (100 days) | RP4 | >8192 | >512 | >64 | ND | ND | ND |
| 23 | D100-E:H:p-3 | A+K  (100 days) | RP4 | >4096 | >256 | >64 | ND | ND | ND |
| 24 | D100-E:H:p-4 | A+K  (100 days) | RP4 | >4096 | >256 | >64 | ND | ND | ND |
| 25 | D100-E:H:p-5 | A+K  (100 days) | RP4 | >4096 | >256 | >64 | ND | ND | ND |
| 26 | The **e**volved A:H:p was treated as the **E**volved bacterial **H**ost with **p**lasmid: E:H:p-1 | A+K  (150 days) | RP4 | >4096 | >256 | >64 | >32 | >4 | >0.06 |
| 27 | E:H:p-2 | A+K  (150 days) | RP4 | >4096 | >256 | >64 | >32 | >4 | >0.06 |
| 28 | E:H:p-3 | A+K  (150 days) | RP4 | >4096 | >256 | >64 | >32 | >4 | >0.06 |
| 29 | E:H:p-4 | A+K  (150 days) | RP4 | >4096 | >256 | >64 | >32 | >4 | >0.06 |
| 30 | E:H:p-5 | A+K  (150 days) | RP4 | >4096 | >256 | >64 | >32 | >4 | >0.06 |
| 31 | E:H:p-6 | A+K  (150 days) | RP4 | >4096 | >256 | >64 | >32 | >4 | >0.06 |
| 32 | E:H:p-7 | A+K  (150 days) | RP4 | >4096 | >256 | >64 | >32 | >4 | >0.06 |
| 33 | E:H:p-8 | A+K  (150 days) | RP4 | >4096 | >256 | >64 | >32 | >4 | >0.06 |
| 34 | E:H:p-9 | A+K  (150 days) | RP4 | >4096 | >256 | >64 | >32 | >4 | >0.06 |
| 35 | E:H:p-10 | A+K  (150 days) | RP4 | >4096 | >256 | >64 | >32 | >4 | >0.06 |
| 36 | E:H:p-11 | A+K  (150 days) | RP4 | >4096 | >256 | >64 | >32 | >4 | >0.06 |
| 37 | E:H:p-12 | A+K  (150 days) | RP4 | >4096 | >256 | >64 | >32 | >4 | >0.06 |
| 38 | E:H:p-13 | A+K  (150 days) | RP4 | >4096 | >256 | >64 | >32 | >4 | >0.06 |
| 39 | E:H:p-14 | A+K  (150 days) | RP4 | >4096 | >256 | >64 | >32 | >4 | >0.06 |
| 40 | E:H:p-15 | A+K  (150 days) | RP4 | >4096 | >256 | >64 | >32 | >4 | >0.06 |
| 41 | E:H:p-16 | A+K  (150 days) | RP4 | >4096 | >256 | >64 | >32 | >4 | >0.06 |
| 42 | E:H:p-17 | A+K  (150 days) | RP4 | >4096 | >256 | >64 | >32 | >4 | >0.06 |
| 43 | E:H:p-18 | A+K  (150 days) | RP4 | >4096 | >256 | >64 | >32 | >4 | >0.06 |
| 44 | E:H:p-19 | A+K  (150 days) | RP4 | >4096 | >256 | >64 | >32 | >4 | >0.06 |
| 45 | E:H:p-20 | A+K  (150 days) | RP4 | >4096 | >256 | >64 | >32 | >4 | >0.06 |
| 46 | E:H:p-21 | A+K  (150 days) | RP4 | >4096 | >256 | >64 | >32 | >4 | >0.06 |
| 47 | E:H:p-22 | A+K  (150 days) | RP4 | >4096 | >256 | >64 | >32 | >4 | >0.06 |
| 48 | E:H:p-23 | A+K  (150 days) | RP4 | >4096 | >256 | >64 | >32 | >4 | >0.06 |
| 49 | E:H:p-24 | A+K  (150 days) | RP4 | >4096 | >256 | >64 | >32 | >4 | >0.06 |
| 50 | E:H:p-25 | A+K  (150 days) | RP4 | >4096 | >256 | >64 | >32 | >4 | >0.06 |
| 51 | **C**ontrol strain of **A**:**H:p** in evolution experiments without antibiotic stress**:**  C:H:p-1 | N  (150 days) | RP4 | >8192 | >512 | >64 | >16 | >1 | 0.01 |
| 52 | C:H:p-2 | N  (150 days) | RP4 | >8192 | >512 | >64 | >16 | >1 | 0.01 |
| 53 | C:H:p-3 | N  (150 days) | RP4 | >8192 | >512 | >64 | >16 | >1 | 0.01 |
| 54 | C:H:p-4 | N  (150 days) | RP4 | >8192 | >512 | >64 | >16 | >1 | 0.01 |
| 55 | C:H:p-5 | N  (150 days) | RP4 | >8192 | >512 | >64 | >16 | >1 | 0.01 |
| 56 | C:H:p-6 | N  (150 days) | RP4 | >8192 | >512 | >64 | >16 | >1 | 0.01 |
| 57 | C:H:p-7 | N  (150 days) | RP4 | >8192 | >512 | >64 | >16 | >1 | 0.01 |
| 58 | C:H:p-8 | N  (150 days) | RP4 | >8192 | >512 | >64 | >16 | >1 | 0.01 |
| 59 | C:H:p-9 | N  (150 days) | RP4 | >8192 | >512 | >64 | >16 | >1 | 0.01 |
| 60 | C:H:p-10 | N  (150 days) | RP4 | >8192 | >512 | >64 | >16 | >1 | 0.01 |
| 61 | C:H:p-11 | N  (150 days) | RP4 | >8192 | >512 | >64 | >16 | >1 | 0.01 |
| 62 | C:H:p-12 | N  (150 days) | RP4 | >8192 | >512 | >64 | >16 | >1 | 0.01 |
| 63 | C:H:p-13 | N  (150 days) | RP4 | >8192 | >512 | >64 | >16 | >1 | 0.01 |
| 64 | C:H:p-14 | N  (150 days) | RP4 | >8192 | >512 | >64 | >16 | >1 | 0.01 |
| 65 | C:H:p-15 | N  (150 days) | RP4 | >8192 | >512 | >64 | >16 | >1 | 0.01 |
| 66 | C:H:p-16 | N  (150 days) | RP4 | >8192 | >512 | >64 | >16 | >1 | 0.01 |
| 67 | C:H:p-17 | N  (150 days) | RP4 | >8192 | >512 | >64 | >16 | >1 | 0.01 |
| 68 | C:H:p-18 | N  (150 days) | RP4 | >8192 | >512 | >64 | >16 | >1 | 0.01 |
| 69 | C:H:p-19 | N  (150 days) | RP4 | >8192 | >512 | >64 | >16 | >1 | 0.01 |
| 70 | C:H:p-20 | N  (150 days) | RP4 | >8192 | >512 | >64 | >16 | >1 | 0.01 |
| 71 | C:H:p-21 | N  (150 days) | RP4 | >8192 | >512 | >64 | >16 | >1 | 0.01 |
| 72 | C:H:p-22 | N  (150 days) | RP4 | >8192 | >512 | >64 | >16 | >1 | 0.01 |
| 73 | C:H:p-23 | N  (150 days) | RP4 | >8192 | >512 | >64 | >16 | >1 | 0.01 |
| 74 | C:H:p-24 | N  (150 days) | RP4 | >8192 | >512 | >64 | >16 | >1 | 0.01 |
| 75 | C:H:p-25 | N  (150 days) | RP4 | >8192 | >512 | >64 | >16 | >1 | 0.01 |
| 76 | **C**ontrol strain of **A**:**H** in evolution experiments without antibiotic stress**:**  C:H-1 | N  (150 days) | n | >4 | >4 | >2 | >16 | >1 | 0.01 |
| 77 | C:H -2 | N  (150 days) | n | >4 | >4 | >2 | >16 | >1 | 0.01 |
| 78 | C:H -3 | N  (150 days) | n | >4 | >4 | >2 | >16 | >1 | 0.01 |
| 79 | C:H-4 | N  (150 days) | n | >4 | >4 | >2 | >16 | >1 | 0.01 |
| 80 | C:H-5 | N  (150 days) | n | >4 | >4 | >2 | >16 | >1 | 0.01 |
| 81 | C:H-6 | N  (150 days) | n | >4 | >4 | >2 | >16 | >1 | 0.01 |
| 82 | C:H-7 | N  (150 days) | n | >4 | >4 | >2 | >16 | >1 | 0.01 |
| 83 | C:H-8 | N  (150 days) | n | >4 | >4 | >2 | >16 | >1 | 0.01 |
| 84 | C:H-9 | N  (150 days) | n | >4 | >4 | >2 | >16 | >1 | 0.01 |
| 85 | C:H-10 | N  (150 days) | n | >4 | >4 | >2 | >16 | >1 | 0.01 |
| 86 | C:H-11 | N  (150 days) | n | >4 | >4 | >2 | >16 | >1 | 0.01 |
| 87 | C:H-12 | N  (150 days) | n | >4 | >4 | >2 | >16 | >1 | 0.01 |
| 88 | C:H-13 | N  (150 days) | n | >4 | >4 | >2 | >16 | >1 | 0.01 |
| 89 | C:H-14 | N  (150 days) | n | >4 | >4 | >2 | >16 | >1 | 0.01 |
| 90 | C:H-15 | N  (150 days) | n | >4 | >4 | >2 | >16 | >1 | 0.01 |
| 91 | C:H-16 | N  (150 days) | n | >4 | >4 | >2 | >16 | >1 | 0.01 |
| 92 | C:H-17 | N  (150 days) | n | >4 | >4 | >2 | >16 | >1 | 0.01 |
| 93 | C:H-18 | N  (150 days) | n | >4 | >4 | >2 | >16 | >1 | 0.01 |
| 94 | C:H-19 | N  (150 days) | n | >4 | >4 | >2 | >16 | >1 | 0.01 |
| 95 | C:H-20 | N  (150 days) | n | >4 | >4 | >2 | >16 | >1 | 0.01 |
| 96 | C:H-21 | N  (150 days) | n | >4 | >4 | >2 | >16 | >1 | 0.01 |
| 97 | C:H-22 | N  (150 days) | n | >4 | >4 | >2 | >16 | >1 | 0.01 |
| 98 | C:H-23 | N  (150 days) | n | >4 | >4 | >2 | >16 | >1 | 0.01 |
| 99 | C:H-24 | N  (150 days) | n | >4 | >4 | >2 | >16 | >1 | 0.01 |
| 100 | C:H-25 | N  (150 days) | n | >4 | >4 | >2 | >16 | >1 | 0.01 |
| Notes: N: without antibiotics; n: without plasmids; ND: not detected.  A+K (150 days)_:_ exposure of 10 mg·L^-1^ ampicillin (A) and 5 mg·L^-1^ kanamycin (K) for 150 days; N (150 days): without antibiotics for 150 days. | | | | | | | | | |

### Table S2. Primers used in the experiment

|  | Genes |  | Sequneces | Length/bp |
| --- | --- | --- | --- | --- |
| plasmid RP4 identification | *OriV* | F | ctcatctgtcaacgccgc | 300 |
|  |  | R | aaccggctatgtcgtgct |  |
|  | *bla_TEM_* | F | ataactacgatacgggagggc | 765 |
|  |  | R | acatttccgtgtcgccctta |  |
|  | *tetA* | F | tgggttctctatatcgggcg | 500 |
|  |  | R | tgggcgagtgaatgcagaat |  |
| SNP located genes verification | *proQ* | F | tcagtgcggaaggtgaag | 548 |
|  |  | R | ttctaatacggtggcatcc |  |
|  | *hcaT* | F | gctcggctatttcacatac | 947 |
|  |  | R | aacggatgacttcgctac |  |
|  | *rpoC* | F | tcgttctgtaatcaccgtag | 1118 |
|  |  | R | tgtagatgctgttgaaggaa |  |
| Plasmid copy determination | *bla_TEM_* | F | ctacgatacgggagggctta | 81 |
|  |  | R | ataaatctggagccggtgag |  |
|  | *dxs* | F | cgagaaactggcgatcctta | 113 |
|  |  | R | cttcatcaagcggtttcaca |  |
| Efflux pump expression | *AcrA* | F | ggatgacaaagtggaaac | 175 |
|  |  | R | ggcttgctggttattatc |  |
|  | *AcrB* | F | ggtgatgttcagttgttc | 192 |
|  |  | R | agcaataatagaggcgtta |  |
|  | *tolC* | F | ttcctccttcaacaacat | 140 |
|  |  | R | catccaacacatcaacaa |  |
|  | *mdtM* | F | gccgaccagttgaatgggta | 155 |
|  |  | R | tcgatgcaggcagcttaaca |  |
|  | *macA* | F | tcatcgccggactgattacg | 174 |
|  |  | R | atcgccaccgacagagtttt |  |
|  | *macB* | F | cgccatccaaaaacaaccgt | 121 |
|  |  | R | aaaataatcgccgctcacgc |  |
|  | *tetA* | F | cggtcttcttcatcatgcaac | 79 |
|  |  | R | gtcccagtgaaagcgatcc |  |
|  | *tetR* | F | ccgaatgcgtatgattctcc | 84 |
|  |  | R | cgctttactggcacttcagc |  |
| genes expression | *tnaA* | F | cgccaagaaagatgcgatgg | 173 |
|  |  | R | cgtcatacagacctaccgcc |  |
|  | *tnaB* | F | tatttacgggatcggcggtg | 129 |
|  |  | R | ataagattgccgccccagac |  |
|  | *fimA* | F | atctaaagccgctgttgcct | 117 |
|  |  | R | tccaggatctgcacaccaac |  |
|  | *fimH* | F | gtgccaattcctcttaccgtt |  |
|  |  | R | tggaataatcgtaccgttgcg |  |
|  | *flu* | F | gcatgaaagcgtcatcggac | 124 |
|  |  | R | tactgcagttgtggctccag |  |
|  | *aphA* | F | ggcttcgtgatgcctgctt | 198 |
|  |  | R | cattcctggccgtggttct |  |
| reference | *idnT* | F | ctgtttagcgaagaggagatgc | 90 |
|  |  | R | acaaacggcggcgatagc |  |
| plasmid | *sacB* | F | cgatgcaaatgattctatcc | 165 |
|  |  | R | ctgtctgatgctgatacg |  |
|  | *traF* | F | ctccgatggaggccggtat | 196 |
|  |  | R | gggaatgccatctgccttga |  |
|  | *trbB* | F | tagaaacggtggccggattc | 225 |
|  |  | R | cggcgcttttaatgacctcg |  |
|  | *trfA* | F | tgatctgctgcttcgtgtgt | 137 |
|  |  | R | tttggcgaagtcgatgacca |  |
|  | *traJ* | F | ctgcttctcttcgatcttc | 181 |
|  |  | R | ccagggctacaaaatcac |  |

### Table S3-1. Genomic variations observed in culture of C:H:p population 1 (mutational effects were predicted by SnpEFF)

| **Gene name** | **Gene ID** | **Annotation** | **Position** | **SNP Variation** | **AA Variation** | **Mutation** | **Effects** |
| --- | --- | --- | --- | --- | --- | --- | --- |
| *gluQ* | b0144 | glutamyl-Q tRNA synthetase | 164546 | A>T |  | upstream | modifier |
| *fhuA* | b0150 | Ferrichrome outer membrane transporter | 168909 | G>A | Asp>Asn | missense_variant | moderate |
| *rrfH* | b0205 | 5S ribosomal RNA | 228675 | C>G |  | upstream | modifier |
| *fadE* | b0221 | acyl-CoA dehydrogenase | 248238 | T>C |  | upstream | modifier |
| *rhsD* | b0497 | Protein RhsD | 523425 | G>A |  | synonymous_variant | low |
| *rhsD* | b0497 | Protein RhsD | 523796 | C>T | Ala>Val | missense_variant | moderate |
| *ybcY* | b0562 | methyltransferase YbcY | 586056 | T>G |  | upstream | modifier |
| *pflB* | b0903 | Formate acetyltransferase 1 | 953278 | C>G | Gln>Glu | missense_variant | moderate |
| *stfR* | b1372 | Side tail fiber protein | 1431760 | C>T |  | synonymous_variant | low |
| *ansP* | b1453 | L-asparagine permease | 1528453 | G>C |  | upstream | modifier |
| *ydcC* | b1460 | transposase YdcC | 1532311 | G>C | Gly>Arg | missense_variant | |
| *ydfN* | b1547 | side tail fiber protein | 1635050 | C>T |  | synonymous_variant | low |
| *yedD* | b1928 | lipoprotein | 2012439 | G>T |  | upstream | modifier |
| *insD* | b1996 | Transposase | 2069735 | C>T |  | synonymous_variant | low |
| *yeeT* | b2003 | polypeptide | 2076969 | C>T |  | synonymous_variant | low |
| *cbtA* | b2005 | cytoskeleton-binding toxin CbtA | 2077907 | T>C |  | synonymous_variant | low |
| *wcaK* | b2045 | colanic acid biosynthesis pyruvyl transferase | 2123083 | T>A |  | upstream | modifier |
| *cpsB* | b2049 | mannose-1-phosphate guanylyltransferase | 2123084 | A>G |  | splice_region_variant&stop_retained_variant | low |
| *dusC* | b2140 | tRNA-dihydrouridine16 synthase | 2233851 | G>A |  | upstream | modifier |
| *yfcQ* | b2334 | putative fimbrial protein | 2455767 | T>C |  | upstream | modifier |
| *kgtP* | b2587 | α-ketoglutarate:H+ symporter | 2730120 | C>T |  | upstream | modifier |
| *rplS* | b2606 | 50S ribosomal subunit protein L19 | 2746274 | A>G |  | upstream | modifier |
| *tdcB* | b3117 | catabolic threonine dehydratase | 3270099 | T>G |  | upstream | modifier |
| *aaeB* | b3240 | aromatic carboxylic acid efflux pump subunit AaeB | 3392120 | G>A |  | upstream | modifier |
| *rrfF* | b3272 | 5S ribosomal RNA | 3424235 | T>G |  | upstream | modifier |
| *rrfF* | b3272 | 5S ribosomal RNA | 3424236 | A>T |  | upstream | modifier |
| *rhsB* | b3482 | rhs element protein RhsB | 3621678 | C>T |  | synonymous_variant | low |
| *hsrA* | b3754 | putative transporter HsrA | 3943677 | T>C |  | upstream | modifier |
| *hsrA* | b3754 | putative transporter HsrA | 3943681 | T>G |  | upstream | modifier |
| *purD* | b4005 | phosphoribosylamine—glycine ligase | 4210306 | G>C |  | upstream | modifier |
| *nrfD* | b4073 | nrfD protein | 4296416 | C>T |  | downstream | modifier |
| *yzfA* | b4223 | predicted protein | 4448310 | G>A |  | upstream | modifier |

### Table S3-2. Genomic variations observed in culture of C:H:p population 2 (mutational effects were predicted by SnpEFF)

| **Gene name** | **Gene ID** | **Annotation** | **Position** | | **SNP Variation** | **AA**  **Variation** | **Mutation** | **Effects** |
| --- | --- | --- | --- | --- | --- | --- | --- | --- |
| *yadC* | b0135 | fimbrial-like protein | 149757 | | C>T |  | synonymous_variant | low |
| *rrfH* | b0205 | 5S ribosomal RNA | 228675 | | C>G |  | upstream | modifier |
| *fadE* | b0221 | acyl-CoA dehydrogenase | 248193 | | G>A |  | upstream | modifier |
| *mhpT* | b0353 | H+ symporter | | 375148 | A>C |  | upstream | modifier |
| *rhsD* | b0497 | protein RhsD | | 524025 | T>G |  | synonymous_variant | low |
| *rhsD* | b0497 | protein RhsD | | 525274 | G>C | Gly>Arg | missense_variant | moderate |
| *ybcY* | b0562 | methyltransferase YbcY | | 586056 | T>G |  | upstream | modifier |
| *rhsC* | b0700 | rhs element protein RhsC | | 732974 | G>A | Ser>Asn | missense_variant | moderate |
| *ybfD* | b0706 | transposase YbfD | | 738400 | A>C | Lys>Asn | missense_variant | moderate |
| *ybfD* | b0706 | transposase YbfD | | 738410 | G>A | Ala>Thr | missense_variant | moderate |
| *ybfD* | b0706 | transposase YbfD | | 738819 | T>C | Val>Ala | missense_variant | moderate |
| *ybgQ* | b0718 | fimbrial usher protein YbgQ | | 749763 | C>A | Leu>Met | missense_variant | moderate |
| *poxB* | b0871 | pyruvate oxidase | | 915199 | T>A |  | upstream | modifier |
| *aspC* | b0928 | aspartate aminotransferase | | 987581 | A>T |  | upstream | modifier |
| *yccE* | b1001 | protein YccE | | 1064201 | G>A | Val>Ile | missense_variant | moderate |
| *ansP* | b1453 | L-asparagine permease | | 1528453 | G>C |  | upstream | modifier |
| *ydfW* | b1567 | Qin prophage | | 1651264 | G>A |  | upstream | modifier |
| *rspA* | b1581 | mandelate racemase | | 1654280 | C>A | Leu>Met | missense_variant | moderate |
| *yeeJ* | b1978 | inverse autotransporter adhesin | | 2050013 | T>A |  | synonymous_variant | low |
| *yeeT* | b2003 | CP4-44 prophage | | 2076891 | T>C |  | synonymous_variant | low |
| *cbtA* | b2005 | CP4-44 prophage | | 2077817 | T>A | Cys>* | stop_gained | high |
| *wcaK* | b2045 | colanic acid biosynthesis pyruvyl transferase | | 2123083 | T>A |  | upstream | modifier |
| *cpsB* | b2049 | mannose-1-phosphate guanylyltransferase | | 2123084 | A>G |  | splice_region_variant&stop_retained_variant | low |
| *ompC* | b2215 | outer membrane porin C | | 2312225 | C>T |  | synonymous_variant | low |
| *yfcQ* | b2334 | fimbrial protein YfcQ | | 2455767 | T>C |  | upstream | modifier |
| *kgtP* | b2587 | H+ symporter | | 2725802 | A>G |  | upstream | modifier |
| *rplS* | b2606 | 50S ribosomal subunit protein L19 | | 2746274 | A>G |  | upstream | modifier |
| *yfjQ* | b2633 | CP4-57 prophage | | 2768847 | C>T |  | synonymous_variant | low |
| *insD-4* | b2860 | IS2 insertion element protein InsB | | 2996927 | A>C |  | synonymous_variant | low |
| *insD-4* | b2860 | IS2 insertion element protein InsB | | 2997041 | A>C |  | synonymous_variant | low |
| *insD-5* | b3045 | IS2 insertion element protein InsB | | 3186648 | A>T | Thr>Ser | missense_variant | moderate |
| *insH-10* | b3218 | IS5 transposase and trans-activator | | 3367775 | T>C |  | upstream | modifier |
| *insH-10* | b3218 | IS5 transposase and trans-activator | | 3367777 | T>G |  | upstream | modifier |
| *aaeB* | b3240 | aromatic carboxylic acid efflux pump subunit AaeB | | 3392120 | G>A |  | upstream | modifier |
| *rbbA* | b3486 | ribosome-associated ATPase | | 3634431 | A>C |  | upstream | modifier |
| *yhiL* | b4660 | DUF4049 domain-containing protein YhiL | | 3639198 | C>A |  | upstream | modifier |
| *hsrA* | b3754 | putative transporter HsrA | | 3943681 | T>G |  | upstream | modifier |
| *nrfD* | b4073 | reductase subunit NrfD | | 4296416 | C>T |  | downstream | modifier |
| *insC-6* | b4272 | IS2 insertion element repressor InsA | | 4498289 | G>A |  | synonymous_variant | low |

### Table S3-3. Genomic variations observed in culture of C:H:p population 3 (mutational effects were predicted by SnpEFF)

| **Gene name** | **Gene ID** | **Annotation** | **Position** | **SNP variants** | **AA variants** | **Mutation** | **Effects** |
| --- | --- | --- | --- | --- | --- | --- | --- |
| *insB-1* | b0021 | Insertion element IS1 | 20120 | T>G | Ser>Arg | missense_variant | moderate |
| *fhuA* | b0150 | fhuA | 168041 | C>T |  | synonymous_variant | low |
| *metQ* | b0197 | lipoprotein MetQ | 225223 | C>T |  | upstream | modifier |
| *fadE* | b0221 | Acyl-coenzyme A dehydrogenase | 248251 | A>C |  | upstream | modifier |
| *insD-1* | b0361 | tRNA ribosyltransferase-isomerase | 382536 | G>A | Arg>Gln | missense_variant | moderate |
| *rhsD* | b0497 | Protein RhsD | 526390 | A>C |  | synonymous_variant | low |
| *ybbD* | b0501 | protein YbbD | 528060 | C>T |  | upstream | modifier |
| *tfaD* | b0561 | Protein TfaD | 581968 | T>G | Asp>Glu | missense_variant | moderate |
| *ybcY* | b0562 | protein YbcY | 586056 | T>G |  | upstream | modifier |
| *ybfD* | b0706 | transposase YbfD | 738426 | T>C | Ile>Thr | missense_variant | moderate |
| *etk* | b0981 | Tyrosine-protein kinase etk | 1042773 | A>C | Thr>Pro | missense_variant | moderate |
| *pinE* | b1158 | Serine recombinase PinE | 1210164 | T>G |  | synonymous_variant | low |
| *ycgV* | b1202 | protein YcgV | 1254006 | C>T |  | synonymous_variant | low |
| *rssB* | b1235 | Regulator of RpoS | 1290924 | A>C | Gln>Pro | missense_variant | moderate |
| *stfR* | b1372 | side tail fiber protein | 1430569 | T>G |  | synonymous_variant | low |
| *insD-2* | b1402 | Transposase InsD for insertion element IS2D | 1468704 | C>T |  | synonymous_variant | low |
| *ansP* | b1453 | L-asparagine permease | 1528558 | G>A |  | upstream | modifier |
| *rzpQ* | b1553 | Qin prophage | 1644442 | C>A |  | upstream | modifier |
| *ynjD* | b1756 | ABC transporter ATP-binding protein | 1839091 | T>A | Asn>Lys | missense_variant | moderate |
| *cheR* | b1884 | methyltransferase | 1968582 | T>G |  | synonymous_variant | low |
| *insD-3* | b1996 | CP4-44 prophage | 2069225 | C>T |  | synonymous_variant | low |
| *galF* | b2042 | glucose-1-phosphate uridylyltransferase | 2118599 | C>T |  | upstream | modifier |
| *wcaK* | b2045 | colanic acid biosynthesis | 2123077 | C>A |  | upstream | modifier |
| *cpsB* | b2049 | mannose-1-phosphate guanylyltransferase | 2123084 | A>G |  | splice_region_variant&stop_retained_variant | low |
| *dusC* | b2140 | tRNA-dihydrouridine synthase | 2233997 | G>A |  | upstream | modifier |
| *aaeB* | b3240 | romatic carboxylic acid efflux pump | 3392120 | G>A |  | upstream | modifier |
| *rrfF* | b3272 | 5S ribosomal RNA | 3424237 | G>A |  | upstream | modifier |
| *rhsB* | b3482 | rhs element protein RhsB | 3619224 | C>T |  | synonymous_variant | low |
| *yhhI* | b3484 | transposase | 3624873 | G>C | Gly>Arg | missense_variant | moderate |
| *gltS* | b3653 | sodium symporter | 3828778 | G>T |  | upstream | modifier |
| *hsrA* | b3754 | transporter HsrA | 3943681 | T>G |  | upstream | modifier |
| *aslB* | b3800 | anaerobic sulfatase maturation enzyme | 3984193 | G>A |  | splice_region_variant&stop_retained_variant | low |
| *yihD* | b3858 | protein YihD | 4040647 | G>A |  | upstream | modifier |
| *yshB* | b4686 | protein YshB | 4056623 | T>A |  | upstream | modifier |
| *purD* | b4005 | phosphoribosylamine—glycine ligase | 4210306 | G>C |  | upstream | modifier |
| *yzfA* | b4223 | Putative YjfA | 4448310 | G>A |  | upstream | modifier |
| *insD-6* | b4273 | Transposase InsD for insertion element IS2K | 4498915 | T>C |  | synonymous_variant | low |

### Table S4. Genomic variations observed in experimental evolution (mutational effects were predicted by SnpEFF)

| **Gene name** | **Gene ID** | **Annotation** | **Position** | **SNP variants** | **AA variants** | **Mutation** | **Effect** | **Population** |
| --- | --- | --- | --- | --- | --- | --- | --- | --- |
|  |  |  |  |  |  |  |  |  |
| *proQ* | b1831 | RNA chaperone ProQ | 1915290 | A>T | Asp>Val | SNP–missense | moderate | E:H:p-1  E:H:p-2  E:H:p-3  E:H:p-4  E:H:p-5 |
| *yeeZ* | b2016 | putative epimerase | 2088564 | C>A | His>Asn | SNP–missense | moderate | E:H:p-2  E:H:p-3  E:H:p-5 |
| *hcaT* | b2536 | putative 3-phenylpropionate transporter | 2667006 | T>A | Trp>Arg | SNP–missense | moderate | E:H:p-1  E:H:p-2  E:H:p-3  E:H:p-4  E:H:p-5 |
| *uxaA* | b3091 | Altronate dehydratase | 3243058 | A>T | Asp>Val | SNP–missense | moderate | E:H:p-1  E:H:p-4 |
| *rpoC* | b3988 | RNA polymerase subunit β' | 4186605 | A>C | His>Pro | SNP–missense | moderate | E:H:p-1  E:H:p-2  E:H:p-3  E:H:p-4  E:H:p-5 |
| synonymous | b3172 | argininosuccinate synthetase | 3319956 | G>A | - | - | - | E:H:p-2 |
| synonymous | b3173 | argininosuccinate synthetase | 3319957 | G>A | - | - | - | E:H:p-5 |
| intergenic | Upstream of lacZ (20 bp),  Downstream of lacI (101 bp) | - | 366326 | A>G | - | - | - | E:H:p-2  E:H:p-4  E:H:p-5 |
|  | Upstream of pyrE (40 bp),  Downstream of rph (53 bp) | - | 3815809 | C>T | - | - | - | E:H:p-1 E:H:p-2  E:H:p-3  E:H:p-5 |
|  | Downstream of gltP (266 bp), Downstream of yjcO (375 bp) | - | 4296060 | C>T | - | - | - | E:H:p-3  E:H:p-4  E:H:p-5 |
| No mutations | N | N | N | N | N | N | | C:H:p-1  C:H:p-2  C:H:p-3  C:H:p-4  C:H:p-5  A:H:p-1  A:H:p-2  A:H:p-3  A:H:p-4  A:H:p-5 |

### Table S5. Genomic variations observed in evolved H strains culture (mutational effects were predicted by SnpEFF)

| **Gene name** | **Gene ID** | **Annotation** | **Position** | **SNP variants** | **AA variants** | **Mutation** | **Effect** | **Population** |
| --- | --- | --- | --- | --- | --- | --- | --- | --- |
| *fhuA* | b0150 | Ferrichrome outer membrane transporter | 168251 | G>A |  | synonymous_variant | low | culture2 |
| *fadE* | b0221 | Acyl-coenzyme A dehydrogenase | 248251 | T>G |  | upstream | modifier | culture1,2 |
| *gnl* | b4580 | Lactonase | 392981 | C>T |  | synonymous_variant | low | culture1 |
| *gnl* | b4580 | Lactonase | 392982 | G>A | Ala>Thr | missense_variant | moderate | culture3 |
| *rhsD* | b0497 | Protein RhsD | 525411 | A>C | Glu>Asp | missense_variant | moderate | culture1,3 |
| *rhsD* | b0497 | Protein RhsD | 526900 | T>G | Tyr>Asp | missense_variant | moderate | culture1,2 |
| *rhsD* | b0497 | Protein RhsD | 526902 | C>T |  | synonymous_variant | low | culture1 |
| *ybcN* | b0547 | protein YbcN | 572789 | C>G |  | synonymous_variant | moderate | culture1,2 |
| *ybcN* | b0547 | protein YbcN | 572792 | C>A |  | synonymous_variant | moderate | culture1,2,3 |
| *rhsC* | b0700 | Protein RhsC | 732811 | A>G | Thr>Ala | missense_variant | moderate | culture1,2 |
| *rhsC* | b0700 | Protein RhsC | 732813 | C>G |  | synonymous_variant | low | culture1,2,3 |
| *rhsC* | b0700 | Protein RhsC | 732828 | T>C |  | synonymous_variant | low | culture1,3 |
| *ybfD* | b0706 | putative transposase YbfD | 738640 | G>T | Lys>Asn | missense_variant | moderate | culture1,2 |
| *lysQ* | b0749 | Probable prophage lysozyme | 781489 | G>A |  | upstream | modifier | culture2 |
| *lysQ* | b0749 | Probable prophage lysozyme | 781504 | A>T |  | upstream | modifier | culture3 |
| *yccE* | b1001 | protein YccE | 1064198 | A>C | Lys>Gln | missense_variant | moderate | culture1,2 |
| *tfaE* | b1156 | Prophage tail fiber assembly protein | 1212090 | C>T |  | upstream | modifier | culture1,3 |
| *yncD* | b1451 | TonB-dependent receptor YncD | 1527958 | T>C |  | upstream | modifier | culture2 |
| *ydcC* | b1460 | putative transposase YdcC | 1531906 | C>T |  | synonymous_variant | low | culture3 |
| *ydcC* | b1460 | putative transposase YdcC | 1531944 | C>T |  | synonymous_variant | low | culture1 |
| *ydfU* | b1560 | protein YdfU | 1643737 | A>C | Leu>Val | missense_variant | moderate | culture2,3 |
| *rspA* | b1581 | Starvation-sensing protein RspA | 1654836 | G>A |  | synonymous_variant | low | culture2,3 |
| *rspA* | b1581 | Starvation-sensing protein RspA | 1654839 | G>T |  | synonymous_variant | low | culture2 |
| *rspA* | b1581 | Starvation-sensing protein RspA | 1654842 | A>C |  | synonymous_variant | low | culture2 |
| *rsxC* | b1629 | Ion-translocating oxidoreductase | 1708862 | C>T |  | synonymous_variant | low | culture3 |
| *flu* | b2000 | antigen 43 | 2072390 | C>G |  | synonymous_variant | low | culture1 |
| *flu* | b2000 | antigen 43 | 2072395 | A>T | Asn>Ile | missense_variant | moderate | culture1,3 |
| *ompC* | b2215 | Outer membrane porin C | 2312057 | A>C |  | synonymous_variant | low | culture1 |
| *ompC* | b2215 | Outer membrane porin C | 2312058 | C>T | Ala>Val | missense_variant | moderate | culture1,2,3 |
| *yfbK* | b2270 | protein YfbK | 2385435 | T>A | His>Gln | missense_variant | moderate | culture1,2 |
| *yfcR* | b2335 | fimbrial-like protein YfcR | 2451581 | T>G | Thr>Pro | missense_variant | moderate | culture2 |
| *luxS* | b2687 | S-ribosylhomocysteine lyase | 2818049 | T>A |  | upstream | modifier | culture1 |
| *hycI* | b2717 | Hydrogenase 3 maturation protease | 2842573 | C>T |  | splice_region_variant&stop_retained_variant | low | culture1 |
| *yhhI* | b3484 | putative transposase YhhI | 3624818 | G>T | Glu>Asp | missense_variant | moderate | culture1,2 |
| *bcsA* | b3533 | Cellulose synthase | 3700139 | T>C |  | upstream | modifier | culture1 |
| *dppD* | b3541 | Dipeptide transport | 3708318 | A>C |  | upstream | modifier | culture2 |
| *pyrE* | b3642 | Orotate phosphoribosyltransferase | 3817618 | ATT>AT |  | upstream | modifier | culture1 |
| *ilvB* | b3671 | Acetolactate synthase | 3856352 | TT>TTAT |  | upstream | modifier | culture1,2 |
| *hsrA* | b3754 | transport protein HsrA | 3943396 | G>A |  | upstream | modifier | culture3 |
| *cpxA* | b3911 | Sensor histidine kinase | 4108724 | T>C |  | upstream | modifier | culture2 |
| *purD* | b4005 | Phosphoribosylamine--glycine ligase | 4210398 | G>A |  | upstream | modifier | culture1 |
| *pyrB* | b4245 | Aspartate carbamoyltransferase | 4477272 | G>T |  | upstream | modifier | culture3 |

In order to obtain the high quality of mutations from bacterial culture, the filter criteria were set to be reads number (5) and mutation quality (>20).

### Table S6. Isolates from murine fecal samples

| **Number** | **isolates** | **Amounts/isolates** |
| --- | --- | --- |
| 1 | E:H:p | 219 |
| 2 | *Escherichia* | 24 |
|  | *Escherichia coli* | 23 |
|  | *Escherichia fergusonii* | 1 |
| 3 | *Burkholderia* | 3 |
|  | *Burkholderia fungorum* | 3 |
| 4 | *Klebsiella* | 4 |
|  | *Klebsiella pneumonia* | 2 |
|  | *Klebsiella singaporensis* | 2 |
| PCR products of 27F and 1496R were sequenced by sanger sequencing, and then were blasted on NCBI database | | |

**References**

1. Sprouffske K,Wagner A. Growthcurver: an R package for obtaining interpretable metrics from microbial growth curves. BMC Bioinformatics. 2016;17:172.

2. Darkoh C, Chappell C, Gonzales C, Okhuysen P. A rapid and specific method for the detection of indole in complex biological samples. Appl Environ Microbiol. 2015;81:8093-7.

3. Merritt JH, Kadouri DE, O'Toole GA. Growing and analyzing static biofilms. Curr Protoc Microbiol. 2005;Chapter 1:Unit 1B

4. Loftie-Eaton W, Bashford K, Quinn H, Dong K, Millstein J, Hunter S, et al. Compensatory mutations improve general permissiveness to antibiotic resistance plasmids. Nat Ecol Evol. 2017;1:1354-63.

5. Hale L, Lazos O, Haines A, Thomas C. An efficient stress-free strategy to displace stable bacterial plasmids. Biotechniques. 2010;48:223-8.

6. Lee HJ, Kim HJ, Lee SJ. CRISPR-Cas9-mediated pinpoint microbial genome editing aided by target-mismatched sgRNAs. Genome Res. 2020;30:768-75.

7. Jiang Y, Chen B, Duan C, Sun B, Yang J, Yang S. Multigene editing in the *Escherichia coli* genome via the CRISPR-Cas9 system. Appl Environ Microbiol. 2015;81:2506-14.
